# Clinicians’ views of factors influencing decision-making for caesarean section: A systematic review and metasynthesis of qualitative, quantitative and mixed methods studies

**DOI:** 10.1371/journal.pone.0200941

**Published:** 2018-07-27

**Authors:** Sunita Panda, Cecily Begley, Deirdre Daly

**Affiliations:** 1 School of Nursing and Midwifery, Trinity College Dublin, Dublin, Ireland; 2 Sahlgrenska Academy, University of Gothenburg, Gothenburg, Sweden; Massachusetts General Hospital, UNITED STATES

## Abstract

**Background:**

Caesarean section rates are increasing worldwide and are a growing concern with limited explanation of the factors that influence the rising trend. Understanding obstetricians’ and midwives’ views can give insight to the problem. This systematic review aimed to offer insight and understanding, through aggregation, summary, synthesis and interpretation of findings from studies that report obstetricians’ and midwives’ views on the factors that influence the decision to perform caesarean section.

**Methods:**

The electronic databases of PubMed (1958–2016), CINAHL (1988–2016), Maternity and Infant Care (1971–2016), PsycINFO (1980–2016) and Web of Science (1991–2016) were searched in September 2016. All quantitative, qualitative and mixed methods studies, published in English, whose aim was to explore obstetricians’ and/or midwives’ views of factors influencing decision-making for caesarean section were included. Papers were independently reviewed by two authors for selection by title, abstract and full text. Thomas *et al*’s 12 assessment criteria checklist (2003) was used to assess methodological quality of the included studies.

**Result:**

The review included 34 studies: 19 quantitative, 14 qualitative, and one using mixed methods, involving 7785 obstetricians and 1197 midwives from 20 countries. Three main themes, each with several subthemes, emerged. Theme 1: “clinicians’ personal beliefs”–(‘Professional philosophies’; ‘beliefs in relation to women’s request for CS’; ‘ambiguous versus clear clinical reasons’); Theme 2: “health care systems”–(‘litigation’; ‘resources’; ‘private versus public/insurance/payments’; ‘guidelines and management policy’). Theme 3: “clinicians’ characteristics” (‘personal convenience’; ‘clinicians’ demographics’; ‘confidence and skills’).

**Conclusion:**

This systematic review and metasynthesis identified clinicians’ personal beliefs as a major factor that influenced the decision to perform caesarean section, further contributed by the influence of factors related to the health care system and clinicians’ characteristics. Obstetricians and midwives are directly involved in the decision to perform a caesarean section, hence their perspectives are vital in understanding various factors that have influence on decision-making for caesarean section. These results can help clinicians identify and acknowledge their role as crucial members in the decision-making process for caesarean section within their organisation, and to develop intervention studies to reduce caesarean section rates in future.

## Introduction

Caesarean section (CS) rates have risen substantially over the past few decades, often without clear rationale [1] and limited additional maternal or neonatal benefits [2]. Maternity care is regarded as being at the core of the health care system. Mode of birth and associated outcomes are widely debated because of the absence of clear reasons, increasing CS rates, and the belief that some CSs are unnecessary, [3] and lack of rationale for the steady rise [1]. Some of the factors contributing to the rise in rates include complexities associated with caring for women with a high body mass index (BMI) or following infertility treatment [4,5,6], however, many factors remain under explored or poorly explained [7]. There are some suggestions that some CSs are performed without medically justifiable reasons [8] or are attributable to women’s choice. However, women’s request has not been identified as a factor to the increase in CS, and a systematic review of literature concluded that only a minority of women (15.6%) in both developing and developed countries expressed their preference to birth by CS [9,10].

Research on clinicians’ views of factors that influence their decision to perform a CS include clinicians’ personal beliefs of what is considered clinical or non-clinical [11], hospital policies/guidelines [12, 13], financial issues and private health care coverage, [14, 15] fear of legal consequences [16, 17], lack of access to facilities and resources [11, 14], and lack of co-operation among professionals [18, 19].

A number of strategies have been identified to reduce the likelihood of performing unnecessary CSs, including the promotion of vaginal birth [20], preparing women for labour and birth, and supporting women during labour. Other strategies at individual clinician level include case selection for induction of labour, involving consultant obstetricians in the decision-making process [21], and avoiding medically unnecessary primary CS [22]. Effective strategies at hospital level include having guidelines and protocols, conducting regular audits [11], using quality control performance charts [23], and having leadership and executive support [24]. However, the reasons and/or factors that influence the decision to perform a CS remain poorly explained and under-explored in most of these studies. Hence, this systematic review and metasynthesis was conducted to ascertain and explore the thoughts, perspectives, views and experiences of clinicians directly involved with the decision-making for CS with the aim of understanding underlying reasons for decisions made and providing an explanation and clarification from the decision-makers’ perspective.

### Aim

To provide insight and understanding, through aggregation, summary, synthesis and interpretation of findings from studies that report obstetricians’ and midwives’ views on the factors that influence the decision-making to perform CS.

## Materials and methods

### Study design

This systematic review and metasynthesis complies with the PRISMA guidelines (S1 Appendix PRISMA Checklist), was modelled on Lucas et al’s (2007) [25] framework and consisted of four successive stages:

- Step 1: Data collection from, and independent review of, studies that met the aim of the research was conducted according to a protocol, developed *a priori*, conducting a scoping search followed by a systematic search of published articles, using the agreed search strategy, and selecting studies that met the aim of the review.
- Step 2: Identification and isolation of emergent themes from the findings of each study by ensuring accuracy and reliability of the final findings with an aim to pool them together to derive the broader themes.
- Step 3: Clustering together of themes to identify broad themes and derive the subthemes to describe and present clinicians’ views of factors that influenced the decision-making for CS in clinicians’ own words.
- Step 4: Synthesis of findings from the studies and describing them under each derived theme and subtheme to address the key issue of ‘factors that influence decision-making for CS, according to obstetricians’ and midwives’ views’.

### Search strategy and selection criteria

The electronic databases of PubMed (1958–2016), CINAHL (1988–2016), Maternity and Infant care (1971–2016), PsycINFO (1980–2016) and Web of Science (1991–2016) were searched in September 2016 for studies that reported obstetricians’ and midwives’ views on the factors that influence the decision to perform a CS.

The PICo (P- Population, I- Interest, Co- Context) approach was used to break down the objectives and underpin the search strategy.

*Population (P)*: The terms to identify Population (P) included the obstetricians and/or midwives (e.g. ‘obstetrician’ OR ‘obstetricians’ AND/ OR ‘midwife’ OR ‘midwives’)

*Interest (I)*: Terms to identify the Interest (I) were related to identifying views and perspectives (e.g., ‘view or views’ OR ‘Perspective or perspectives’) of the participants.

*Context (Co)*: Terms to identify the Context (Co) included factors influencing decision-making for CS (e.g. ‘decision-making’ AND ‘caesarean section’ or ‘caesarean section’ or ‘factors’).

Search terms were combined using the Boolean operand ‘AND’ (for example ‘caesarean section’ AND ‘clinicians’ AND ‘views’), using the key words *‘caesarean section’*, *‘midwives’*, *‘obstetricians’*, *‘views’*, *‘factors’* etc. (S2 Appendix presents the complete search strategy).

This systematic review included all quantitative, qualitative, and mixed methods studies published in the English language that reported on the views of obstetricians and midwives on the factors that influence the decision to perform a primary (first-time) and/or repeat CS. Studies reporting only on women’s views or women's experiences (despite their recognised importance), and views of other health care professionals such as hospital administrators, policy makers, anaesthetists, neonatologists, etc were excluded in order to focus on obstetricians’ and midwives’ views, for this paper.

### Study selection

Retrieved papers were reviewed independently by two independent authors by title and abstract (by SP and DD), then by full text (by SP & DD; and SP & CB). Two authors had to agree included papers, and any disagreements were discussed with the third author.

### Assessment of methodological quality of included studies

A modified version of Thomas *et al’s* (2003) [26] 12-point quality assessment criteria checklist was used to assess the methodological quality of included studies (S3 Appendix) because it facilitated assessment of quantitative, qualitative and mixed methods studies. Each criterion on the tool was scored ‘1’ if the criterion was met, or ‘0’ if not met. Studies were assessed and scored under three categories, and the total scores were categorised as ‘weak’ (Scores 0–6), ‘moderate’ (Scores 7–9) OR ‘strong’ (Scores 10–12) methodological quality. A decision was made *a priori* to exclude studies that scored ‘6’ or less. Three authors (SP, DD and CB) independently assessed the methodological quality of included papers, and confirmed the final score and each paper’s inclusion for data extraction.

### Data extraction

A data extraction tool was developed to report a full description of each study, including design, participants and sample size, data collection method(s), data analysis and findings reported by the author(s).

### Data analysis

Thematic analysis of each included study’s findings was conducted using Lucas *et al*’s (2007) framework [25]. Themes from individual studies were pooled and clustered into emerging broader themes and subthemes, then synthesised. Meta-analysis planned in advance was not possible because there was considerable heterogeneity between the quantitative studies (different populations and different surveys used), so a thematic analysis and meta-synthesis was performed.

## Findings and results

### Study selection

The search retrieved 1463 individual titles, resulting in 1098 studies after removing the duplicates (n = 365) (Table 1).

**Table 1:** Results of search strategy for each database.

Data base	Dates	Results
PubMed	01/01/1958–30/09/2016	812
CINAHL	01/01/1988–30/09/2016	158
Maternity and Infant Care	01/01/1971–30/09/2016	393
PsycINFO	01/01/1980–30/09/2016	89
Web of Science	01/01/1991–30/09/2016	11

A total of 918 studies were excluded by ‘title and abstract’ (reviewed by two independent authors (SP&DD), leaving 180 studies for full text review. Each full text paper was independently reviewed by two authors (SP & DD; and SP & CB) and a total of 127 studies were excluded because they did not report on clinicians’ views of factors influencing decision-making for CS (S1 Fig).

### Results of assessment of methodological quality

The 53 included papers were reviewed for assessment of methodological quality by all three authors (SP, DD and CB). Of these, nineteen papers scored ‘6’ or less, and were excluded from final analysis, leaving 34 papers for data extraction and analysis. The excluded papers (n = 19) had lower scores, mainly, in relation to reliability and validity of data collection tools, methods of data collection and data analysis. The remaining 34 papers scored as moderate (score 7 to 9) (n = 19) or high quality (score 10–12) (n = 15) (S4 Appendix).

### Study characteristics

The 34 studies were published during the 24-year period from 1992 to 2016. The included studies involved 7785 obstetricians (in 33 studies) and 1197 midwives (in 11 studies); only one study included a combination of obstetricians and midwives (n = 26) [13]. The studies were conducted in 20 countries; 23 were conducted in 12 OECD countries (http://www.oecd.org/countries/) and 11 studies in eight non-OECD countries (S5 Appendix). As the focus was on clinicians’ views, views of other personnel, family planning workers (n = 18) [27]; hospital administrator (n = 1) [17]; insurance bodies, syndicates and scientific societies, ministries, international agencies, medical schools, media representatives and women’s groups (n = 20) [28] and professional decision-makers (n = 9) [11] were excluded from data extraction and analysis. Obstetricians in Huang *et al*’s (2013) study [27] were described as ‘township doctors’ who were involved in the decision to perform CS, and have therefore been included for analysis.

Nineteen studies used a quantitative design (surveys, postal questionnaires), 14 used qualitative designs (individual or focus group interviews) and one used mixed methods design (interviews and surveys). Table 2 presents the summary characteristics of the included studies.

**Table 2:** Summary characteristics of the studies.

Author/ Year/ Country	Aim		Study design	Participants and sample size	Data collection	Data analysis	Key findings reported by author(s)
Appleton *et al* (2000) [29] Australia	To establish the level of knowledge and the background attitudes of staff towards VBAC		Survey	159 consultant obstetricians and 116 registrars/ residents 681 midwives (RR = 67%)	Questionnaires	Chi-square analysis	**Obstetricians:** Previous classical caesarean, **breech** and twins. **Parental anxiety** was a major factor influencing a decision for or against a trial of labour. **Midwives:** Previous classical caesarean, **Midwives perceived** higher risk associated with trial of labour.
Arikan *et al* (2011) [15] Turkey	(1) To investigate the caesarean rate among actively practicing obstetricians in Turkey and reasons why they choose this mode of delivery for themselves /partners. (2) To investigate the attitudes, practices, and beliefs with respect to caesarean delivery on maternal request (CDMR) among actively practicing obstetricians in Turkey.		Descriptive	387 obstetricians (RR = 77%)	Self-administered questionnaire	Chi-square, Mann–Whitney U, and Kruskal–Wallis tests	**Obstetricians:** Most common reason for choosing CS was reduced **ano-rectal trauma**. CS on maternal request. **Private hospitals** with significantly higher rate of CS due to maternal request compared to public hospitals
Bagheri *et al* (2013) [14] Iran	To explore obstetricians' views of what might influence pregnant women's choice of delivery		Qualitative	18 obstetricians	Semi-structured Interview	Inductive qualitative content analysis	**Obstetricians:** Women’s **right and previous experience**. Personal preferences for CS, **shortage of midwives**, **lack of cooperation** between midwives and obstetricians. **Fear of litigation**. CS believed to be **safer than vaginal birth**.
Bailit *et al* (2007) [30] United States	To determine which primary caesarean delivery risk factors are important to practising obstetricians		Survey	259 obstetricians (RR = 29%)	Questionnaire	Wilcoxon signed rank test	**Obstetricians: Medical problems, maternal obesity,** macrosomic infant, malpresentation, Bishop score, **patient's fear**
Bergholt *et al* (2004) [31] Denmark	To assess Danish obstetricians’ and gynaecologists’ personal preference and general attitude towards elective caesarean section on maternal request in uncomplicated single cephalic pregnancies at term.		Survey	364 obstetrician and gynaecologists (RR = 80%)	Questionnaire	Multiple logistic regression analysis	**Obstetricians: Risk to the fetus, risks of perineal injury. Woman’s right to have an elective caesarean section** on maternal request without any medical indication.
Bettes *et al* (2007) [16] United States	To examine obstetrician–gynecologists’ knowledge, opinions, and practice patterns related to caesarean delivery on maternal request.		Survey	699 obstetricians and gynaecologists (591 of these were involved in conducting births) (RR = 68%)	Questionnaire	Descriptive statistics, independent sample t tests, 2-test	**Obstetricians:** No **policy regarding CS on maternal request**. **Media portrayal** on CS. **Difference in views** between male and female obstetricians. **Women’s right** to request for CS. The **risk of urinary and fecal incontinence and pelvic floor** prolapse. **Convenience. Liability concerns**.
Bryant *et al* (2007) [18] Australia	To explore the beliefs through which decisions for caesarean birth are made and to consider how this might contribute to the increasing rate of caesarean birth		Qualitative	6 obstetricians 12 hospital based midwives	Interviews	Thematic analysis	**Obstetricians: Women’s right** to choose CS. **Risks associated with** CS is viewed as minimal. **Powerful belief systems among obstetricians**. Midwives: **Midwives contested** the notion of free choice. **Maternal request in absence of medical indication.**
Chaillet *et al* (2007) [32] Canada	To investigate obstetricians’ perceptions of clinical practice guidelines targeting management of labour and vaginal birth after previous caesarean birth, and to identify the barriers to, facilitators of and obstetricians’ solutions for implementing these guidelines in practice.		Qualitative	27 obstetricians	Focus group and individual interviews	Thematic analysis	**Obstetricians:** Management and hospital policy; **medicolegal concerns**, **skill levels**, acceptance of guidelines, **nature of medical explanations** provided, and the management of maternal request for medical interventions.
Chalmers *et al* (1992) [33] South Africa	To investigate doctors' perceptions of CS practices and explore the availability of facilities which could help to reduce the high CS rate		Survey	203 obstetricians (RR = 45.2%)	Questionnaire	Chi-square analysis	**Obstetricians:** Reasons for first CS are **Dystocia, fetal distress,** etc. Marked **difference between private and hospital based doctors** with private doctors more readily performing CS compared to hospital based doctors. **Fear of litigation**, **financial incentives** for high CS rates.
Chigbu *et al* (2010) [34] Nigeria	to determine obstetricians’ attitude to and factors predicting obstetricians’ acceptance of caesarean delivery on maternal request in Nigeria		Survey	211 obstetricians (RR = 70.3%)	Questionnaire	Multiple logistic regression analysis	**Obstetricians:** Positive attitude of obstetricians' to maternal autonomy and maternal request for CS. No influence of obstetricians' bio-professional characteristics on CS.
Coleman *et al* (2005) [35] United States	To assess obstetrician-gynaecologists' current practice patterns and opinions regarding vaginal birth after caesarean delivery (VBAC)		Survey	502 obstetricians and gynaecologists (RR = 41.8%)	Questionnaire	Descriptive statistics, t- test, Chi square test and Spearman analysis	**Obstetricians: Multifetal gestation, diabetes and obesity**. P**atient preference and risk of liability**. High repeat CS reported by **private physicians compared to physicians working in not-for-profit hospitals.**
Coleman-Cowger *et al* (2010) [36] United States	To determine obstetricians-gynaecologists' practice patterns of caesarean delivery on maternal request (CDMR) following the 2006 National Institutes of Health (NIH) State-of-the-Science Conference on this topic, and compare them with those in their practice prior to the conference		Survey	352 obstetricians and gynaecologists (RR = 59%)	Questionnaire	Descriptive statistics, t- tests, Chi square test and Wilcoxon Signed Ranks test, power analysis	**Obstetricians:** Significant agreement to the statement that **woman has a right to request and obtain an elective CS**. M**aternal age, plans for future childbearing**, week of pregnancy, BMI, fetal size, maternal anxiety
Colomar *et al* (2014) [11] United States	To explore attitudes of physicians attending births in the public and private sectors and at the managerial level toward caesarean birth in Nicaragua		Qualitative-descriptive	17 obstetricians and gynaecologists	Individual and focus group interviews	Descriptive analysis	**Obstetricians:** F**etal weight, presentation, history of previous birth by CS**, **breech.** Obstetricians were not aware of **existing standards**. **Defensive medicine and lack of guidelines**. Lack of human and material **resources, Convenience**
Cotzias *et al* (2001) [37] United Kingdom	To determine what proportion of obstetricians would agree to elective pre-labour CS for 'maternal request'		Survey	151 consultant obstetricians (RR = 61.4%)	Questionnaire	Descriptive analysis	**Obstetricians:** Obstetricians agree to **maternal request** for CS in absence of medical indication in case of a women who is well informed of the risks associated with CS. Common reason is **litigation**
Cox, K. J. (2011) [17] United States	To explore the barriers associated with the ACOG VBAC guidelines, as well as the strategies that obstetricians and midwives use to minimize their legal risks when offering a trial of labor after caesarean		Qualitative	11 obstetricians and 12 midwives	Semi-structured Interview	Thematic analysis	**Obstetricians: Fear of liability and restrictions from hospital policies** and consulting physicians. **Convenience**. Lack of availability of anaesthetist. **Financial benefits** **Midwives: Fear of liability. Convenience**. Exclusion of midwives from **policy-making**.
Danishevski *et al* (2008) [38] Russia	To identify the factors that Russian obstetricians take into account when recommending a Caesarean section		Qualitative—Interviews	92 practising obstetricians (Response rate is not reported in the paper)	Responses to vignettes	Conjoint analysis	**Obstetricians: Birth weight** of 3.5 kgs or more, **gestation of over 42 weeks, maternal age** of 32 years or above, time of the day, **male obstetricians** were three times **more likely** to recommend CS compared to female obstetricians.
Doret *et al* (2010) [39] France	To evaluate obstetricians’ practice patterns, opinions and factors influencing decision-making about mode of delivery in women with two previous c-sections		Survey	105 obstetricians (RR = 65.6%)	Questionnaire	Non-parametric Mann-Whitney test or *t* test, Chi square test.	**Obstetricians:** Factors that negatively influence VBAC following two previous CSs were **increased maternal and neonatal risks** and VBAC not being a **standard of care** for these women.
Faas-Fehervary *et al* (2005) [40] Germany	To evaluate the influence of biographic data, working environment and personal birth experience on the attitude towards Caesarean Section on demand.		Survey	719 gynaecologists (RR = 34%)	Questionnaire	Chi square and *t*-test	**Obstetricians:** Approval for CS on demand is related to **patient autonomy** and physicians' **age, personal birth experiences**
Foureur *et al* (2016) [41] Australia	To explore the views and experiences of providers in caring for women considering VBAC, in particular the decision-making processes and the communication of risk and safety to women.		Qualitative Descriptive interpretive	3 obstetricians and 15 midwives	Focus group interviews	Thematic analysis	**Obstetricians:** Clinicians’ p**ositive orientation** towards VBAC. **Midwifery care** was viewed as integral to achieve VBAC. **Different perspectives** among midwives and obstetricians. Midwives: **Positive orientation** towards VBAC. **Midwives did not express fears concerning the risks** of VBAC.
Fuglenes and Kristiansen (2009) [42] Norway	The aim of this study was to test the hypothesis that obstetricians’ choice of delivery method is influenced by their risk attitude and perceived risk of complaints and malpractice litigation		Survey	507 obstetricians (RR = 71%)	Questionnaire—5 clinical scenarios presented	Chi square test for bivariate analysis of categorical variables and t-test for continuous ones. Logistic regression	**Obstetricians:** Perceived risk of complaints and malpractice **litigation** were two clear determinants for the choice of CS by obstetricians. **Maternal request** is a driving force leading to higher CS rates.
Huang *et al* (2013) [27] China	To assess population-based caesarean section (CS) rates in rural China and explore determinants and reasons for choosing a CS		Qualitative	24 township doctors	Focus group interviews	Frame work approach was used for analysis	**Obstetricians:** CS less time consuming, confidence of obstetrician. Financial benefit to the hospital. **Maternal request**
Josefsson *et al* (2011) [43] Sweden	To compare Swedish obstetricians'/gynecologists' and midwives’ attitudes and opinions on different aspects of caesarean section (CS)		Survey	846 obstetricians 278 midwives (RR = 66%)	Questionnaire	Chi square test and student's *t*-test	**Obstetricians: Difference in attitudes of midwives and obstetricians about rates of CS.** **Midwives: Difference in attitudes of midwives and obstetricians**
Kabakian-Khasholian *et al* (2007) [28] Lebanon	This study aims to provide an analysis of the policy environment encouraging C-section in Beruit and its suburbs and to reveal approaches that could be adopted for the reduction of this practice, by considering the attitudes, opinions and actions of different stakeholders.		Qualitative	10 obstetricians	Interview and group discussions	Applied political analysis	**Obstetricians: Lack of skilled obstetricians, convenience**, lack of unified **standards and guidelines**, **maternal demands** for CS, diversity in medical education. **Women's request**. Lack of facilities. **Private insurance**,
Kamal *et al* (2005) [12] United Kingdom	To explore the views of health professionals on the factors influencing repeat caesarean section.		Qualitative	12 doctors and 13 midwives (6 hospital-based and 7 community midwives)	Semi-structured interviews	Constant compar-ative method	**Obstetricians: Repeat CS** was a major contribution. F**etal distress, breech presentation, poor fetal growth, preeclampsia.** Avoiding subsequent **litigation.** **Midwives:** Repeat CS for women who had previous birth by CS **and breech presentation**. **Lack of 'quality of evidence', Professional boundaries.** Avoiding subsequent **litigation**.
Karlstrom *et al* (2009) [44] Sweden	To describe obstetricians’ and midwives’ attitudes towards CS on maternal request.		Qualitative	9 obstetricians and 16 midwives	Focus group discussions	Content analysis. Themes were derived	**Obstetricians: Previous negative birth experience,** fear related to child birth, hospital working condition, **fear of litigation**. Presence of a midwife could enhance positive birth experience. Midwives: **Heavy work load**, **stress in intrapartum care. Fear of litigation**.
Kenton *et al* (2005) [45] United States	To determine the practice patterns and opinions of recently trained US obstetrician-gynaecologists regarding repeat CS, primary elective CS, and elective CS for the prevention of pelvic floor disorders		Survey	304 obstetrician-gynaecologists (RR = 61%)	Questionnaire	Mann-Whitney and McNemar tests, Chi square test	**Obstetricians:** Lack of availability of anaesthesia facility. Risks of **uterine rupture, neonatal morbidity/mortality issues, haemorrhage, preventing pelvic floor.**
Koigi-Kamau *et al* (2005) [46] Kenya	To determine perceptions, preferences and practices of vaginal birth after Caesarean.		Survey	64 obstetricians in private practice (RR = 60%)	Questionnaire	Descriptive statistics	**Obstetricians:** Increased **demand for repeat** by women, obstetricians' **convenience, fear of litigation** in case of complications.
Kwee *et al* (2004) [47] Netherlands	To determine the opinion of Dutch gynaecologists and registrars on caesarean section (CS) on request		Survey	583 gynaecologists and registrars (RR = 65%)	Questionnaire	Analysis of variance and logistic regression analysis	**Obstetricians: Autonomy for the woman**, **litigation**. Influence of obstetricians', gender and experience on decision to perform CS.
Litorp, *et al* (2015a) [48] Tanzania	To explore women's and caregivers' experiences, perceptions, attitudes, and beliefs in relation to caesarean section.		Qualitative	18 obstetricians and 8 midwives	Individual and focus group interviews, and participant observations.	Thematic analysis	**Obstetricians: Women's low level of education**. Care providers believed that **vaginal birth is unpredictable**. Socio-economic consequences for women. Midwives: Vaginal birth is unpredictable.
Litorp *et al* (2015b) [19] Tanzania	To explore obstetric care givers' rationales for their hospital's CS rate to identify factors that might cause CS overuse.		Qualitative	18 obstetricians and 14 midwives	Individual and focus group interviews	Thematic analysis	**Obstetricians: Conflict and difference** in attitude. Lack of resources. **Maternal age and weight. Private patients'** request for CS. **Litigation** Midwives: **Conflict and difference** in attitude among professionals. Lack of resources (equipments, staff shortages). **Litigation**
Monari *et al* (2008) [49] Italy	To explore the attitudes toward caesarean section of midwives and obstetricians who worked in the same geographical area		Survey	100 obstetricians and 148 midwives (public sector only) (RR = 94.6%)	Structured questionnaire	Fisher's extract and Chi square tests	**Obstetricians: Reduce the chances of stress and fecal incontinence**. **Difference in attitudes**. **Male obstetricians were more likely to agree to or perform CS than females.** **Midwives:** Risks associated with CS. **Medico legal problems**
Samadi *et al* (2013) [50] Iran	To assess the behaviour and preferred delivery method among Iranian obstetricians in challenging cases.		Survey	75 obstetricians (Response rate is not reported in the paper)	Revised Jackson personality inventory questionnaire	Prevalence of response and risk scores	**Obstetricians: Medicolegal issues**, avoiding risks
Weaver and Richards (2007) [51] United Kingdom	To examine whether, and in what context, maternal requests for caesarean section are made		Mixed methods	29 obstetricians (interviews) and 785 consultants (questionnaires) (RR = 58%)	Survey and Interviews	Using SPSS (for surveys) and thematic analysis	**Obstetricians: Maternal request**, **fear of litigation** and defensive medicine
Yazdizadeh *et al* (2011) [13] Iran	To identify barriers to reduce the caesarean section rate in Iran, as perceived by obstetricians and midwives as the main behavioural change target groups.		Qualitative	26 obstetricians and midwives (number of midwives and obstetricians are not presented separately in the published article)	In-depth interviews	Thematic analysis	**Obstetricians: Financial** and **judicial** problems. **Absence of on call physician**. Shortage resources. **Distrust and insufficient collaborations Medicalisation** of labour. **Absence of hospital protocol** **Midwives:** The **type and ownership of hospitals.** Shortage of **human resources** and facilities at the Distrust and insufficient collaborations between obstetricians and midwives. **Absence of hospital protocol**

## Thematic analysis and meta-synthesis

A tabular summary of the findings of each study was constructed, and then findings from all studies were compared, contrasted, aggregated, integrated and synthesised to derive the themes. It also allowed comparing and contrasting the findings reported in each study. Findings are presented using clinicians’ own words, and are not an interpretation of the clinicians’ views. Findings included clinicians’ perspectives of a range of factors in relation to the decision to perform all types of CS including primary and repeat CSs. Thematic analysis resulted in the emergence of three interrelated key themes; ‘clinicians’ personal beliefs’, ‘health care systems’ and ‘clinicians’ characteristics’. While each theme is of equal importance, ‘clinicians’ personal beliefs’ emerged as the driver of the decision to perform CS. Table 3 presents the themes and subthemes reported in each study. S2 Fig—Diagrammatic presentation of the themes and subthemes (34 studies)

**Table 3:** Themes and subthemes presented in each study.

Author/Year	Theme 1: Clinicians' personal beliefs	Theme 2: Health care systems	Theme 3: Clinicians ‘ characteristics
	Subtheme 1.i Professional philosophies- a. Perception of risk; b. ‘CS’ being a safe option; c. Lack of cooperation and trust Subtheme 1.ii Beliefs in relation to women's request for CS Subtheme 1.iii Ambiguous versus clear clinical reasons	Subtheme 2.i. Litigation; Subtheme 2.ii. Resources; Subtheme 2.iii. Private versus public/insurance/ payments; Subtheme 2.iv. Guidelines and management policy	Subtheme 3.i. Personal convenience; Subtheme 3.ii. Clinicians’ demographics; Subtheme 3.iii. Confidence and skills
**Appleton *et al* (2000) [29]**	1.i.a; 1.ii; 1.iii	2.iv	3.ii
**Arikan *et al* (2011) [15]**	1.i.a; 1.i.b; 1.ii; 1.iii	2. iii.	
**Bagheri *et al* (2013) [14]**	1.i.a; 1.i.b; 1.i.c; 1.ii	2.i; 2.ii; 2.iii	
**Bailit *et al* (2007) [30]**	1.iii		
**Bergholt *et al* (2004) [31]**	1.ii; 1.iii		
**Bettes *et al* (2007) [16]**	1.ii; 1.iii	2.i; 2.iv	3.i
**Bryant *et al* (2007) [18]**	1.i.a; 1.i.b; 1.i.c; 1.ii		3.i
**Chaillet *et al* (2007) [32]**	1.ii	2.i; 2.ii; 2.iv	3.iii
**Chalmers *et al* (1992) [33]**	1.i.a; 1.i.b; 1.ii; 1.iii	2.i; 2.ii; 2.iii	3.i
**Chigbu *et al* (2010) [34]**	1.ii	2.i	3.ii
**Coleman *et al* (2005) [35]**	1.ii; 1.iii	2.i; 2.ii; 2.iii	
**Coleman-Cowger *et al* (2010) [36]**	1.ii; 1.iii		
**Colomar *et al* (2014) [11]**	1.i.a; 1.i.b; 1.ii; 1.iii	2.i; 2.ii; 2.iv	3.i
**Cotzias *et al* (2001) [37]**	1.ii	2.i	
**Cox (2011) [17]**	1.i.a; 1.i.c	2.i; 2.ii; 2.iv	3.i
**Danishevski *et al* (2008) [38]**	1.iii	2.ii	3.ii
**Doret *et al* (2010) [39]**	1.ii; 1.iii	2.iv	
**Faas-Fehervary *et al* (2005) [40]**	1.i.b	2.i; 2.iii	3.ii
**Foureur *et al* (2016) [41]**	1.i.a; 1.i.b	2.i	
**Fuglenes and Kristiansen (2009) [42]**	1.ii	2.i	3.ii
**Huang *et al* (2013) [27]**	1.ii	2.iii	3.i; 3.iii
**Josefsson *et al* (2011) [43]**	1.i.c; 1.ii		
**Kabakian-Khasholian *et al* (2007) [28]**	1.ii	2.iii; 2.iv	3.i; 3.iii
**Kamal *et al* (2005) [12]**	1.i.a; 1.ii; 1.iii	2.i; 2.ii; 2.iv	
**Karlstrom *et al* (2009) [44]**	1.i.a; 1.i.b; 1.ii	2.i; 2.ii	3.i
**Kenton *et al* (2005) [45]**	1.iii	2.ii	3.ii
**Koigi-Kamau** **and Kiarie (2005) [46]**	1.ii	2.i	3.i
**Kwee *et al* (2004) [47]**	1.i.b; 1.ii	2.i	3.ii
**Litorp *et al* (2015a) [48]**	1.i.a; 1.i.b; 1.ii		
**Litorp *et al* (2015b) [19]**	1.i.a; 1.i.c; 1.ii; 1.iii	2.i; 2.ii.	
**Monari *et al* (2008) [49]**	1.i.a; 1.iii	2.i	3.ii
**Samadi *et al* (2013) [50]**	1.i.a; 1.i.b	2.i	
**Weaver and Richards (2007) [51]**	1.ii; 1.iii	2.i	
**Yazdizadeh *et al* (2011) [13]**	1.i.a; 1.i.b; 1.i.c; 1.ii	2.i; 2.ii; 2.iii.; 2.iv	3.i

### Theme 1: Clinicians’ personal beliefs

Clinicians’ personal beliefs and their influence on the decision to perform CS were discussed in all 34 included studies, and three interlinked subthemes were identified; “professional philosophies”, “beliefs in relation to women’s request for CS” and “ambiguous versus clear clinical reasons”. All these three subthemes were interlinked, with “clinicians’ personal beliefs” emerging as the key driver.

#### Subtheme 1.i: Professional philosophies

Obstetricians’ own philosophies around decision-making for CS were reported in 18 studies, with many including references to the attitudes of their obstetric and midwifery colleagues. These mostly included agreements or disagreements with clinicians’ perception of risk associated with CS/vaginal birth/VBAC (15 studies), their personal preferences and a perception of CS being a ‘safe option’ (12 studies) and lack of co-operation and trust among professionals (6 studies) (Table 3).

**1.i.a. Perception of risk:** Obstetricians’ and midwives’ perceptions of risk associated with CS was reported in 15 studies as a factor influencing the (choice of) mode of birth, mostly attributable to risks for the mother and fetus, and a general perception that some degree of risk was associated with CS compared to vaginal birth (first birth or VBAC) [13, 14, 17, 18, 41, 44, 50]. More than half of the Iranian obstetricians (53% (n = 40)) in Samadi *et al*’s study chose CS to avoid any risk in unclear situations [50]. Similarly, almost half (48%, n = 335) of the obstetricians in Bettes et al’s study (2007) in the United States (US) performed CS because of perceived concerns related to the risk of urinary and fecal incontinence and pelvic floor prolapse following vaginal births [16].

*“But some of the times when you go into caesars*, *and you see how paper thin that lower segment is*, *it’s terrifying…if you have contractions on that*, *your chances of beating it* [the lower segment]*, it just goes” (Obstetrician) (Foureur et al, 2016.p.3) [41]*

*‘‘Previously, when I was resident, we used to say, at first we should save the mother, and she can have another pregnancy later, but now the life of the newborn is as important as the life of mother. We can’t give a dead child to the mother … So if there can be a least possible risk for the fetus, we choose CS” (obstetrician) (Bagheri et al, 2013*.*p.48) [14]*

In absence of any medical indication, midwives in Litorp *et al*’s study regarded vaginal birth as preferable to CS; however in general, they had a positive attitude towards CS [48].

*“… In general*, *I think it* [CS] *is good … It's good because it helps mothers to enjoy the fruits of pregnancy” (Midwife) (Litorp et al, 2015a p.716) [48]*

**1.i.b. CS being a ‘safe option’:** Clinicians’ personal preferences and beliefs about CS (reported in 12 studies) were mostly dependent on what they perceived as safe. Obstetricians perceived CS to be a safe option [48] because they believed that it could reduce the risks of and prevent complications for women who lived in isolated areas with lack of access to facilities [11]. In Arikan et al’s (2011) study obstetricians acknowledged some of the complications associated with CS such as infection, adhesions and complications of anaesthesia, etc., but about two-third of them preferred CS as the mode of birth for themselves or their partners [15].

*“Earlier on, CS was very dangerous in our setting. Nowadays that we feel that CS is safe, we tend to do more CSs.” (Senior obstetrician) (Litorp et al, 2015a p*.*717) [48]*

Compared to vaginal births, obstetricians often viewed CS as a safe option due to its reduced risk of complications (Bagheri et al, 2013). Sometimes elective CS was considered as a safer and better option than an emergency CS [18].

*“Elective caesarean sections I view as being quite safe. Emergency caesarean sections, because you’re rushing, and may be … a bit more dangerous, although still it’s a relatively safe operation.” (Obstetrician) (Bryant et al, 2007 p*.*1197) [18]*

Midwives often perceived obstetricians’ belief of ‘CS being a safe option’, as one of the factors that influenced the decision to perform CS [18].

*“You know all that kind of talk around*, *“it’s the most dangerous journey the baby will ever make*, *down the women’s vagina*.*” And*, *so they’ve lost faith*, *some of them* …*I actually think that the belief system amongst obstetricians is now that it’s* [CS] *so safe that why would you risk that whole painful, messy, vaginal, risky business?” (Midwife) (Bryant et al, 2007 p.1197) [18]*

*“It depends on the doctor that they get and how it’s put to them* [women]. *Because sometimes they* [women] *go in* [to the antenatal visit with the doctor] *going*, *‘‘I’m not sure*. *I’m not sure”*. *They come back with*, *‘‘No*, *I want a caesarean*. *Because it’s* [a VBAC] *not safe.” (Midwife FGD) (Foureur et al, 2016 p.4) [41]*

**1.i.c. Lack of cooperation and trust:** A lack of cooperation and trust between obstetricians and midwives, as well as between obstetricians with different levels of expertise (residents/registrars and specialists/consultants), were identified as influencing factors in six studies.

Residents in Litorp *et al*’s study (2015b) reported a negative view around the decision-making process in their hospital, with specialists not being supportive of the residents, and midwives not being supportive of vaginal births [19].

*“Sometimes you can be called by a midwife and … she … thinks that ‘this woman has to go for CS’. And… you see that there is no good reason. Now you enter into some sort of friction and conflict. . . .You might enter into a situation of decision of unnecessary CS because of … friction with the midwives.” (Resident (obstetric registrar)) (Litorp et al, 2015b p*.*235) [19]*

In Yazdizadeh *et al*’s study (2011), midwives viewed their lack of involvement in the decision-making process as a reason for the rise in CSs [13]; however, a different perspective to this view was reported by Tanzanian midwives, who, despite their belief that their profession was not recognised, felt that they influenced the decision-making process, and made the obstetric residents (registrars) perform a CS, for genuine indications [19].

*“Many times they follow what we tell them. When these junior doctors come in, they come with an attitude … When we tell them stuff, they pretend they know this and that. Those who listen to us, things usually go well for them. The stubborn ones get very bad outcomes. This is why they later change and cooperate with us” (Midwife) (Litorp et al, 2015b, p*.*235) [19]*

*“The discrepancy between the midwives’ and the specialists’ information is our main problem. We don’t believe in issues that the physicians accept as true. We do our best to make physicians accept our proposals in certain cases but the residents change frequently before winning our trust. In other words, too much time is needed before the physicians would accept our proposals and therefore we have to work gradually. We, however, do our best.”(Midwife) (Yazdizadeh et al, 2011, p*. *10) [13]*

Some of the issues in relation to lack of cooperation were related to obstetricians’ perception of the midwife’s role [13, 14].

*“The midwives are a great help, and they are better in vaginal deliveries, but they should take responsibility. If they start the delivery, and then call us in a very serious condition and put the responsibilities to us, I prefer to have a delivery from the very beginning myself.” (Obstetrician) (Bagheri et al, 2013 p*. *47) [14]*

#### Subtheme 1.ii: Beliefs in relation to women’s request for CS

Twenty-six studies reported on clinicians’ views of “women’s request for CS” as an influencing factor. Decision-making was mostly influenced by socio-cultural perspectives, women’s preferences, demands, obstetricians’ beliefs in women’s right and autonomy to choose a CS, and their perception of women’s anxiety and fear.

Maternal request for CS was cited by 77% (n = 604) of the obstetricians in the UK and Ireland as one of the main reasons to perform CS. [51] Similarly, most of the obstetricians (n = 154, 69%) in Cotzias *et al*’s study (2001) agreed to maternal request for CS, and, considered ‘patient pressure’ (n = 55, 89%) and litigation (n = 22, 35%) as the most common factors influencing their decision [37]. Increased demand for a repeat CS (n = 15, 45.7%) and fear of litigation (n = 10, 26.8%) were also reported by Kenyan obstetricians as two reasons for the declining trend of VBAC [46]. More obstetricians than midwives in a Swedish study had a positive attitude towards maternal request for elective CS [43]. Over half of the obstetricians in a US study (n = 322, 54.6%) [16], and over one-third of the obstetricians and gynaecologists in studies in Turkey (n = 158, 40.8%) [15] and Denmark (n = 137, 37.6%) [31] believed that women had a right to choose a CS, and agreed to perform one following discussion of the risks and consequences. In Bettes *et al*’s study, 92.2% (n = 545) of the obstetricians said no policy existed on managing women’s request for CS, but the remaining of the obstetricians 7.8% (n = 46) said a policy existed; and 72.2% (n = 33) of the obstetricians when a policy existed said that it supported women’s request for CS [16]. Other obstetricians and midwives disagreed about women’s preference, right to choice and request for CS.

*“I tell them all the advantages and disadvantages and a complication of caesarean section, but this is the mother, who should choose the type of delivery, although most of the patients are not ready for making the decisions and accept the consequences.” (Obstetrician) (Bagheri et al, 2013 p*.*46) [[14]*

*“At the end of the day, I feel very strongly that women, at the end of the day it’s their body and it’s their right to choose. And I certainly feel that as long as it’s an informed consent, I would be very agreeable to obliging either way.” (Obstetrician) (Bryant et al, 2007 p*.*1194) [18]*

*“I think it’s very fraught. . . .and I don’t think it’s as simple as saying, this is the pros and cons of the situation, now you choose.” (Midwife) (Bryant et al, 2007 p*. *1195) [18]*

Lack of hospital policy and lack of uniformity in following the existing policy and guidelines about managing maternal request for CS was another factor that influenced the decision to perform a CS based on maternal request [16].

Clinicians perceived “women’s anxiety and fear of labour” as one of the most common reasons to request CS in the absence of a medical indication, and obstetricians, in general, favoured these requests [15, 44]. Over half of the US obstetricians (n = 699, 54.6%), attributed the women’s request for CS, mainly, to complications from previous birth (83.9%), and maternal anxiety (71.4%) [16].

*“There are a lot of women who are afraid of everything. They have no trust in their bodily functions or that we are made to give birth.” (Focus group discussion with midwives and obstetricians) (Karlstrom et al, 2009 p*. *60) [44]*

*“Natural birth is painful. Sometimes they have pain for 24 hours … Some have negative experiences from their previous deliveries. They might have a difficult one … When we tell them that second delivery is much easier they don’t believe us, and if we resist, they go to another doctor.” (Obstetrician) (Bagheri et al, 2013 p*.*46) [14]*

Lack of preparedness for labour and birth was reported as one of the other reasons for maternal request for CS [13].

*“…when I meet women in the delivery unit, not all of them know what’s it’s about; they haven’t had the opportunity to practice relaxation. Nobody told them about it.” (Focus group discussion with midwives and obstetricians) (Karlstorm et al, 2009 p*. *60) [44]*

Clinicians believed women’s higher social class, their country’s culture and changes in women’s life style as some of the possible reasons why women requested a CS [13, 14].

*“The ordinary people believe that if someone has a normal delivery that is because she doesn’t have enough money or her husband doesn’t want to spend money for her. They say clearly that we have money, and we pay for caesarean section.” (Obstetrician) (Bagheri et al, 2013 p*. *47) [14]*

*“The modern lifestyle and the anatomical differences between Iranians and those from other countries have affected the former group’s capabilities to undergo vaginal delivery. The sedentary lifestyle and not following a healthy diet have reduced the capabilities of our girls in this regard.” (Obstetrician) (Yazdizadeh et al, 2011 p*. *10) [13]*

Clinicians often viewed the ‘media’ as playing some role in influencing women’s attitude and contributed to the decision to perform CS [13, 44, 50].

*“There have been a lot of writings in the papers and women demand their right to CS*. *There have been a lot in the media*. *In a way it* [caesarean section] *is something you are entitled to, so I believe it’s a lot influenced from there.” (Focus group discussion with midwives and obstetricians) (Karlstrom et al, 2009 p. 60) [44]*

#### Subtheme 1.iii: Ambiguous versus clear clinical reasons

The term ‘ambiguous clinical reasons’ was used to indicate a reason that could, in some clinical situations, be an indication to perform a CS, but not in other circumstances, and was reported in 16 studies. These mostly included maternal reasons such as previous CS [11], risk of anorectal trauma, preventing perineal injury, urinary and anal incontinence [15, 31, 45], maternal age, obesity, previous birth complications [30, 36], risk of pelvic prolapse, uterine rupture [39, 45], medical conditions such as myopia and previous abortions etc. [39]. About two-thirds of the Turkish obstetricians (63%, n = 152) performed a CS to reduce anorectal trauma [15], yet at the same time, many recognised it as not a definite indication for CS.

Breech presentation was also one of the clinical reasons in most of these studies [11, 12, 29, 30, 33] which may or may not be justifiable. The other common reason for performing CS were previous classical CS [29], fetal distress [12], malpresentation [11, 12, 29, 30, 33] dystocia [11, 33], placenta previa [33] and umbilical cord prolapsed [11].

*“We are running high because we are giving caesarean section for a lot more indications now than we used to*. *For instance we used to deliver breeches* [vaginally] *and we no longer deliver breeches* [vaginally]*.” (Obstetrician) (Kamal et al, 2005 p. 1056) [12]*

Although some of these factors were interrelated, there was a degree of ambiguity and uncertainty about performing CS for the above reasons alone, and the final decision, mostly, was influenced by obstetricians’ personal beliefs.

*“Often there’s a slight medical reason in it, such as some people have had a difficult …last time round and may ask for one this time, it may have just been an awful experience, or they may have had a tear and had problems. It’s often difficult to separate them completely” (Obstetric Registrar) (Weaver et al, 2007.p*. *37) [51]*

Reasons reported by midwives mostly included previous CS (Kamal *et al*, 2005), risk of fetal distress and obstructed labour [19].

*“… obviously the fact that somebody had had a previous caesarean section, you are concerned about uterine scar and it’s the sustainability during labour.” (Midwife). (Kamal et al, 2005, p*.*1056) [12]*

### Theme 2: Health care systems

The influence of the aspects of the health care systems were reported in 28 studies. Four subthemes emerged; ‘litigation’, ‘resources’, ‘private versus public/insurance/payments’ and ‘guidelines and management policy’.

#### Subthemes 2.i. Litigation

Clinicians’ fear of litigation was the most common factor influencing the decision to perform a CS in 21 studies.

Most of obstetricians’ fear centred on liability concerns; however, midwives perceived that transferring the responsibility to women in situations of ambiguous cases and in situations with uncertainty surrounding the benefits of vaginal birth was one way obstetricians attempted to avoid subsequent litigation [12]. Midwives confirmed that their practice did not change on the basis of any fear of legal consequences [17].

*“I just think it’s a bunch of crap that you have to change your practice when you know something is safe because somebody might sue you. Anytime you get a less than optimal outcome, people want to blame, people want to sue … It’s just kind of a personal philosophy, too. I just think that most long-term midwives get to that point. Otherwise you’d be too afraid to do anything. Birth is amazing, and not always predictable.” (Midwife) (Cox 2011, p*. *5) [17]*

Many obstetricians, on the other hand, described the medicolegal problems as leaving them with a negative experience [17], a fear and a social stigma [13] and a fear of blame [19] which ultimately, influenced their practice [11].

“[The] *number one priority …is the fear of medico-legal problems because we didn’t do a caesarean, because there is always the probability that a patient may be upset and file a medico-legal complaint.” (Obstetrician) (Colomar et al, 2014 p.2385) [11]*

*“If you have a problem* [during a trial of labour]*, you are going to get no sympathy from the medico-legal community… And nobody is going to be sympathetic for any unusual pattern on the monitor …you can’t tell me of a single VBAC that resulted in a ruptured uterus that wasn’t a disaster medico-legally.” (Obstetrician) (Cox 2011. P.5) [17]*

Just one study, in Nigeria, reported that obstetricians perceived the threat of litigation to be greater after performing CS because women believed that complications arising from natural vaginal birth were unavoidable [34]. In Weaver *et al*’s study (2007), litigation was cited as one of the main reasons to perform CS by 67% (n = 525) of obstetricians in UK and Ireland [51].

#### Subtheme 2.ii: Resources

Eleven studies found that lack of resources influenced the decision to perform CS.

Not having enough experienced clinicians to facilitate a natural birth [14, 32, 44] not only influenced the decision to perform CS, but also led to stress and dissatisfaction among women, and this in turn led to women requesting a CS in subsequent pregnancies [44].

*“You should have a midwife for every woman, now we have a midwife for two or sometimes more than that. So we can’t monitor patients properly. If we have a drop in fetal heart rate, we can’t stay to see what happens. We choose caesarean section very fast” (Obstetrician) (Bagheri et al, 2013. P*. *47) [14]*

*“The major rise in the CS rate in Sweden is due to stress in the delivery units. The women get worried and the doctors are inexperienced.....and intervene too early though it’s a normal process. That’s the main reason….” (FGD with midwives and obstetricians) (Karlstrom et al, 2009, p*.*60) [44]*

Immediate availability of personnel for emergency CS (n = 477, 95%) and/or immediate availability of anaesthesia (n = 462, 92%) were viewed as other important factors that influenced US obstetricians’ decision to choose the mode of birth for women with previous CS [35].

*“The absence of specialists* [consultant obstetricians] *in teaching hospitals is another problem*. *Residents* [obstetric registrars], *who perform the job, decide in favour of C-section as soon as even a small problem is encountered …A skilled physician should always be available in the hospital.” (Obstetrician) (Yazdizadeh et al, 2011. P.7) [13]*

Lack of access to basic infrastructure, including labour rooms, and the condition of the labour environment, were viewed as hindering safe and effective care for women in labour, leading to a reduced rate of normal birth, and ultimately influencing the rate of CSs [12, 13, 44].

*“Contrary to international standards, the size of our labor rooms have reduced and they have been converted into operating rooms over time.” (Midwife) (Yazdizadeh et al, 2011, p*. *9) [13]*

A lack of access to emergency care facilities, such as access to theatre, and lack of availability of labour rooms, were viewed as inhibiting the provision of effective care to women in labour, and influencing the decision to perform CS, especially in remote areas [11, 12, 13, 32, 33, 41]. In Chalmer *et al*’s study, 15% (n = 35 of 233) of obstetricians stated lack of access to facilities influenced their decision to perform CS [33].

*“You must have. . . .a hospital willing to have an operating room ready, a blood bank with the units ordered, that will be ready in 10 minutes. For example, no constraints, don’t allow them to make excuses such as we have no forceps, we don’t provide such services, etc.” (Obstetrician) (Colomar et al, 2014. P*. *2388) [11]*

#### Subtheme 2.iii: Private versus public/ insurance/ payments

The type of health care coverage, hospital (private or public) alone or combined with financial benefits to the institution, emerged as influencing factors in the decision to perform CS in eight studies. South African obstetricians working in private hospitals regarded CS to be a safe option compared to public hospitals based obstetricians [33]. Obstetricians working in private sectors in Turkey reported they had a more positive attitude towards CS on maternal request, and agreed that their CS rates, as a result of maternal requests, are significantly higher compared to obstetricians working in public hospitals [15].

Along with the financial benefits to hospitals and obstetricians, these studies also reported on clinicians’ perception of indirect role played by insurance companies in the decision-making process. Financial incentives were associated with CS, a lower tariff for vaginal births, no reimbursement for epidurals by private insurance companies for vaginal births, which were viewed to be some of the factors influencing the decision to perform CS [11, 13, 14].

*“In the private sector, providers are reimbursed approximately $700 for normal childbirth and $1500 for caesarean section, so the doctor prefers to perform caesarean.” (Obstetricians) (Colomar et al, 2014. P*.*2388) [11]*

#### Subtheme 2.iv: Guidelines and management policy

Nine studies reported on the direct and indirect role played by hospital policies on the decision-making process for performing CS. The lack of unified protocols or guidelines for the management of labour, VBAC, or CS on maternal request (reported by 92% (n = 643) obstetricians in an Australian study) [29], and existing policies supporting CS on maternal request [16, 17] and/or obstetricians being unaware of the existing guidelines and protocols [11] were reported to influence the decision to perform CS.

*“There were…some obstetrics groups that also supported that- they weren’t offering VBAC* [Vaginal Birth After CS] *and didn’t have any desire to consider offering that service. So current hospital policy is that we’re not able to offer a VBAC.” (Obstetrician) (Cox, 2011. p.6) [17]*

In addition, organisations with ‘softer’ criteria for induction of labour, restricted rules for caring for women with previous CS, policies not taking into account women’s individual needs, including over-medicalisation of labour [13, 32], were also perceived as contributing to the decision to perform a CS.

*“…protocols are written in a way, in an absolute sense but do not take into account the normal biological variations that occurs between people and nor do they take into account the interpersonal relationship that exists between the patient and the doctor …”(Obstetrician) (Kamal et al, 2005. p*.*1056) [12]*

Midwives perceived their lack of involvement in the policy-making process, and restrictive rules for care of women with previous CS, as factors that influenced the rise of CS [17].

*“We were doing VBACs with no problem in the hospital, and then, the doctors dropped their malpractice insurance, and we weren’t able to do VBACs, even with the doctors there. Even if the woman wanted the midwives to be giving the care.” (Midwife) (Cox 2011, p*.*7) [17]*

Lack of guidelines or unified protocols for management of labour were some more barriers in facilitating vaginal births [12], ultimately contributing to the decision-making for CS. Midwives, especially community midwives, did not perceive clinical governance as being relevant to their practice, since they are not the primary decision-makers [12].

*“I think you have got to have more clinical guidelines so say ‘yes you can have a caesarean section or not’. But in some cases, in emergency situations, you have got to use your own initiative and leave it to the professional.” (Midwife) (Kamal et al, 2005 p*.*1056) [12]*

### Theme 3: Clinicians’ characteristics

A total of 19 studies identified clinicians’ characteristics as influencing the decision to perform CS, and three subthemes were identified; ‘personal convenience’, ‘clinicians’ demographics’, and ‘confidence and skills’.

#### Subtheme 3.i. Personal convenience

Ten studies reported ‘personal convenience’ as one of the factors influencing the decision to perform a CS. In general, most of the reasons were related to obstetricians’ perception of CS being an ‘organised and controlled’ option and attempts at vaginal birth being ‘highly disordered’ compared to the ‘ease of making an appointment’ for CS [14, 16, 18, 28]. In Bettes *et al*’s study (2007) 23% (n = 136) of the obstetricians perceived scheduling of CS as a convenient option [16]. Obstetricians in Cox’s study (2011) perceived CS to be convenient for two reasons; first reason was women preferring to avoid labour and appreciating the convenient and controlled option in repeat CS; and second reason was having to be available throughout a ‘trial of labour’ imposed significant lifestyle limitations for obstetricians, particularly in rural areas, and midwives in the study reported that obstetricians performed CSs frequently due to its convenience [17].

*“It is certainly easier to do a repeat C-section, so why not just say, ‘Shoot, I don’t have to deal with VBACs, great…and I get to have a little bit of easier life.’ I think when you get to the heart of it, that’s what’s going on.” (Obstetrician) (Cox, 2011. p*.*6) [17]*

*“We should manage our work. The caesarean section gives us the opportunity to manage our schedules, finding someone to work instead of us, tell the hospital when we are leaving. Of course, physicians welcome this”. (Obstetrician) (Bagheri et al, 2013 p*.*e47) [14]*

*“With CS I minimize my time and I earn more*!*” (Obstetrician) (Litorp et al, 2015b) [19]*

*“I have been appalled at how many OBS* [Obstetricians] *will let them pick the date on their first OB visit for their repeat caesarean. Repeat caesareans are not only OK here, they are promoted! They can pick the date, which is very convenient …and they’re selling, they’re selling caesareans.”(Midwife) (Cox, 2011. p.6) [17]*

#### Subtheme 3.ii. Clinicians’ demographics

This subtheme reported in eight studies, included obstetricians’ views of the influence of their personal demographics, such as age, gender and professional status on the decision-making for CS. Three of these studies reported no influence of obstetricians’ demographics [34, 42, 45], and the remaining five studies reported some influence [29, 38, 40, 47, 49].

**Age:** Two studies reported conflicting findings on age as an influential factor [38, 40]. In relation to approving CS on demand, one German study found high rates (70%) of CS among younger obstetricians compared to rates (56%) among older obstetricians [40]; whereas the other study, in Russia, identified a 4% increased risk of approving and performing CS with increasing age of the obstetricians [38].

**Gender:** Three studies that explored the influence of gender, found that male obstetricians were more willing to perform CS than their female colleagues [47, 49, 38]. In the Russian study, male obstetricians were three times more likely to recommend a CS compared to their female colleagues [38].

**Professional status:** Two studies showed that the risk of performing or approving a CS increased with seniority and experience of obstetricians [47, 29]. In a study in the Netherlands, consultants (more than registrars) and experienced doctors (more than less experienced doctors) performed CS more frequently [47], similar to findings from an Australian study where obstetric residents/registrars (83%, n = 116) encouraged ‘trial of labour’ more than consultants or senior colleagues (60%, n = 159) [29].

#### Subtheme 3.iii. Confidence and skills

Clinicians’ decision to perform CS was influenced by a lack of confidence and skills to perform vaginal birth; and mostly they were related to fear of complications, obstetricians’ confidence and skills in facilitating assisted vaginal births and midwives’ confidence and skills in promoting normal births, and this was discussed in three studies [27, 28, 32].

*“Obstetricians are familiar with the operation. Combined with a shortage of skilled midwives and the doctors’ poor skills to attend to a vaginal delivery and manage dystocia, CS may not cause more morbidity or mortality for women and babies than a normal delivery.” (Township doctor (Obstetricians)) (Huang et al, 2013 p*.*917) [27]*.

## Discussion

This systematic review and thematic analysis offers evidence related to clinicians’ views of factors influencing the decision to perform CS, through a synthesis of findings from 34 studies. Three prominent themes, “clinicians’ personal beliefs”, “health care systems” and “clinicians’ characteristics”, emerged as the factors that influenced the clinicians’ decision-making for CS with “clinicians’ personal beliefs” as the key driver.

Although multiple factors influenced clinicians’ decision to perform CS, litigation, women’s request for CS, interprofessional disagreements and private health care and payment systems, are the most common. However, there was a wide difference in interpretation of these factors. Some interpreted it as their personal belief, some saw it in terms of issues related to the health care system and a few attributed it to clinicians’ characteristics. Although maternal request for CS has been a topic of debate over years, recent studies report low rates among nulliparous women, and rates of CS as being unrelated to women’s preferences [10].

Maternal age, BMI, previous CS, etc [29, 30] influenced some clinicians’ decision to perform a CS, but not others, with ambiguity in clinical reasoning to perform a CS. Decision-making was further influenced by clinicians’ perception of the small degree of risk involved in performing a CS and their belief in CS being a ‘safe’ procedure compared to vaginal birth [18].

Midwives and obstetricians in two studies perceived vaginal birth to be the safer and preferred mode of birth including VBAC [12, 41]. Clinicians in most studies reported interprofessional conflict, differences in attitudes, and lack of cooperation as factors that influenced the decision to perform a CS or aim for VBAC [13, 14, 17, 18, 19, 43]. Most of the differences in views among midwives and obstetricians related to their approval or disapproval of CS on maternal request [18]. Clinicians’ perceived women requested for CS for varied reasons including fear of labour [27, 44], previous experience [51], social-culture factors, media and body image, etc [13, 44, 48, 51]. However, obstetricians more so than midwives were inclined to agree to women’s request for CS [12, 15, 37, 43].

Clinicians from OECD and Non-OECD countries had similarities and differences in their views (S6 Appendix: Issues within a cultural context (similarities and differences)—OECD versus Non-OECD countries). While “health care systems” was one of the key themes, the issues and findings within each subtheme differed by geographical, institutional and cultural context. Most of the differences in perspectives related to litigation, human and infrastructural resources, insurance/payment, and private versus public health care system. Clinicians in non-OECD countries were more fearful about the pressure from women, health system and court of law and the resulting stigma [13, 14], whereas fear of complications and adverse outcomes, and being sued in a court of law were some major concerns among clinicians from the OECD countries [41, 42]. In non-OECD countries, issues related mostly to lack of access to and availability of infrastructural resources [19, 33], whereas, in OECD countries issues related to human resources, workload and stress experienced in providing intrapartum care [11, 32]. Insurance, payment systems, and financial benefits to obstetricians emerged as influencing factors from clinicians in non-OECD countries [13, 14], whereas, the influencing factors in OECD countries related to differences in private versus public health care practice [15, 35]. Despite these differences, clinicians from OECD and Non-OECD countries had some similar views about women’s right to choose their mode of birth [14, 18, 44, 45], and CS being a safe [18, 48] and convenient option for child birth [14, 17].

In general, the influence of private health care systems was mentioned frequently by clinicians, sometimes in association with financial, payments or benefits to the hospital [13, 15, 27, 28, 33].

Lack of hospital guidelines or clinicians’ unawareness of the existing guidelines and protocols were other factors that influenced the decision-making process [11, 12, 13, 16, 17, 28, 29, 32].

Fear of legal consequences has been reported previously as an influencing factor for CS [52] as has the contribution of private health care systems [53, 54, 55]. This metasynthesis supported the major and significant influence of ‘litigation’, from the clinicians’ perspectives, on the decision to perform CS, irrespective of the practice setting, age, gender, professional experience, resources and culture within the health care system. Most of the perceived fear related to legal consequences arising from complications associated with vaginal birth compared with birth by CS. There was a perceived pressure from the health care system, court of law, women and their families, which influenced the decision to perform a CS or aim for VBAC [11, 12, 17].

Although not a major factor, clinicians’ characteristics influenced the decision to perform CS. Male obstetricians were more likely, and willing, to perform a CS than their female colleagues [38, 47, 49], and experienced obstetricians (consultants) were perceived to perform CS more frequently than less experienced ones [29, 47].

The ‘personal convenience of performing CS’ emerged as another factor that influenced the obstetricians’ decision to perform CS, or aim for VBAC, and was attributed to or related to obstetricians’ perception of CS being an organised, orderly, convenient and controlled birthing option compared to attempts at vaginal birth and having to be available throughout a trial of labour [14, 16, 17, 18, 28]. Midwives’ perspectives differed and they viewed ‘convenience’ as a means of promoting unnecessary CSs [17].

Clinicians’ decision-making was further influenced by their level of experience, confidence and skills not only in promoting vaginal birth or performing VBACs, but also in managing difficult vaginal births and dealing with any complications which were frequently associated with fear of legal consequences [27, 28, 32]. In one study these factors were attributed to the lack of unified education and training systems for midwives and obstetricians, and gaining skills/experience in managing difficult vaginal births and complications arising [13].

## Conclusion and implications

Obstetricians as final decision-makers for CS are vital determinants of the overall rate of CS in any country. However, many times the factors that influence their decision to perform a CS are multifactorial and complex. This systematic review and metasynthesis identified the range of factors that influence clinicians’ decisions to perform a CS, which include personal, cultural, institutional, legal and financial factors. One of the main key factors that influenced decision-making for CS was ‘clinicians’ beliefs’. This was mostly related to clinicians’ personal preferences, perception of the degree of risk associated with vaginal birth or VBAC, and CS as being a safe and convenient option. Decision-making was influenced further by professional agreements and disagreements among obstetricians and midwives, and obstetricians with different level of experience, clinicians’ fear of litigation, lack of access to manpower and physical resources. Lack of unified guidelines, financial benefits to the hospital, and private versus public health care facilities were all influencing factors, ultimately contributing to the rise in rate of CS.

The rising rate of CS worldwide, particularly for first-time mothers, is a growing concern with lack of evidence related to the factors that influence decision-making. This systematic review and metasynthesis has reduced the gap in information related to some of the complexities associated with the decision-making process from the perspectives of midwives and obstetricians. It has offered insight into the ‘why’ behind the factors influencing rising rate of CS, despite the considerable evidence that vaginal birth is safer and associated with fewer complications compared to birth by CS. Regarding the findings in relation to ‘clinicians' personal beliefs’, ‘health care system issues’ and ‘clinicians’ characteristics’, careful consideration is required to tease out the factors that can be possibly avoided within the maternity care system, to help stop the rise of CS.

This paper presents the first systematic review and synthesis of evidence around clinicians’ perception of factors that influence the decision to perform a CS. Hence, this review will be of significant benefit to policy-makers to revise the institutional policy to aim at improving and promoting normal births and avoiding any unnecessary CSs. It is recommended that care providers give careful consideration to the influencing factors identified here in order to use them to modify their practice, with the aim of reducing CS rates. Further research is recommended to establish how some of the factors identified can be addressed to avoid unnecessary CSs.

This study is limited to the views of clinicians (midwives and obstetricians) whose decision may be further influenced by the health care system, management policy or policy makers. However, the strengths of this metasynthesis lie in its in-depth exploration of the issues, factors and complexities influencing the decision to perform CS from obstetricians’ and midwives’ perspectives. It describes the views of 9008 clinicians from 20 countries in relation to the influence of cultural context, personal philosophies, litigation, women’s request for CS, private health care system and financial issues, and access to resources on their decision to perform CS.

The thematic analysis allowed for in-depth understanding and integration of evidence from both quantitative and qualitative studies, making it a comprehensive presentation of clinicians’ views of complexities associated with the factors that influence the decision to perform a CS. The findings will also help to develop future intervention studies focusing on individual modifiable factors aimed at reducing unnecessary CSs in future.

## Supporting information

S1 Appendix PRISMA checklist. (DOCX)

S2 Appendix Search strategy. (DOCX)

S3 Appendix Tool for assessment of methodological quality. (DOCX)

S4 Appendix Results of assessment of methodological quality. (DOCX)

S5 Appendix OECD and Non-OECD countries. (DOCX)

S6 Appendix Factors—OECD versus Non-OECD countries. (DOCX)

S1 Fig PRISMA flow chart presenting search results. (TIF)

S2 Fig Diagrammatic presentation of the themes and subthemes. (TIF)

## Abbreviations

CS: Caesarean Section
VBAC: Vaginal Birth After Caesarean Section
FGD: Focus Group Discussion
