# Correction: Clinicians' views of factors influencing decision-making for caesarean section: A systematic review and metasynthesis of qualitative, quantitative and mixed methods studies

**DOI:** 10.1371/journal.pone.0202688

**Published:** 2018-08-15

**Authors:** Sunita Panda, Cecily Begley, Deirdre Daly

The Funding section is incorrect. The correct funding information is: This study was funded by Health Research Board, Ireland grant HPF-2016-1671 to SP.
