# Supplementary material for: Clinicians’ views of factors influencing decision-making for caesarean section: A systematic review and metasynthesis of qualitative, quantitative and mixed methods studies
Source: PLoS One. 2018 Jul 27;13(7):e0200941. doi: 10.1371/journal.pone.0200941 (PMC6063415; doi:10.1371/journal.pone.0200941)
Supplement: S2 Appendix — (DOCX) [file pone.0200941.s002.docx]

**S2 Appendix – Search strategy**

**Search string for PubMed**

“cesarean section” OR “cesarean sections” OR “caesarean section” OR “caesarean sections” OR “caesarean delivery” OR “caesarean deliveries” OR “cesarean delivery” OR “cesarean deliveries” OR “caesarian delivery” OR “caesarian deliveries” OR “caesarean birth” OR “caesarean births” OR “cesarean birth” OR “cesarean births” OR “caesarian birth” OR “caesarian births” OR “abdominal delivery” OR “abdominal deliveries” OR “surgical birth” OR “surgical births” OR “surgical birthing” OR cesarean OR cesareans OR caesarean OR caesareans OR caesarian OR caesarians OR LSCS OR “C-Section” OR “C-Sections” OR “C Section” OR “C Sections” OR “medicalized childbirth” OR “medicalized childbirths” OR “medicalised childbirth” OR “medicalised childbirths”

AND

clinician OR clinicians OR midwife OR midwives OR obstetrician OR obstetricians OR “obstetric-nurse” OR “obstetric-nurses” OR “obstetric nurse” OR “obstetric nurses” OR “Nurse Midwives” OR “Nurse Midwife” OR “Nurse-Midwife” OR “Nurse-Midwives” OR “care provider” OR “care providers” OR “health care provider” OR “health care providers” OR professional OR professionals OR physician OR physicians OR doctor OR doctors OR “obstetric consultant” OR “obstetric consultants” OR “consultant obstetrician” OR “consultant obstetricians” OR “clinical manager” OR “clinical managers” OR “midwife manager” OR “midwife managers” OR “midwife-manager” OR “midwife-managers” OR “nurse manager” OR “nurse managers” OR “nurse-manager” OR “nurse-managers” OR “certified professional midwife” OR “certified professional midwives” OR “certified nurse-midwife” OR “certified nurse-midwives” OR “certified midwife” OR “certified midwives” OR “consultant midwife” OR “consultant midwives” OR “advanced midwife practitioner” OR “advanced midwife practitioners” OR “clinical midwife specialist” OR “clinical midwife specialists”

AND

experience OR experiences OR experienced OR view OR views OR viewpoint OR viewpoints OR perception OR perceptions OR perceive OR perceived OR attitude OR attitudes OR belief OR beliefs OR perspective OR perspectives OR opinion OR opinions OR concept OR concepts OR thought OR thoughts OR intuition OR awareness OR comprehension OR value OR values OR understanding

AND

(attitude of health personnel[Mesh Terms] OR attitude to health[Mesh Terms] OR choice behavior[Mesh Terms] OR communication[Mesh Terms] OR consumer participation[Mesh Terms] OR cooperative behavior[Mesh Terms] OR decision making[Mesh Terms] OR decision support techniques[Mesh Terms] OR decision theory[Mesh Terms] OR educational technology[Mesh Terms] OR health education[Mesh Terms] OR informed consent[Mesh Terms] OR professional-family relations[Mesh Terms] OR psychology [Subheading] OR affective aspect* OR choice behavio* OR clinical support technique* OR cognitive aspect* OR collaboration* OR communication* OR compliant behavio* OR consensus OR consent* OR consumer* OR participation* OR cooperative behavio* OR co-operative behavio* OR decision* OR disput* OR dissent* OR doctor patient relationship OR doctor patient relationships OR doctor-patient relationship OR doctor-patient relationships OR educational technology OR emotional aspect* OR health attitude* OR health education OR health information OR health literacy OR illness behavio* OR informed assent OR informed choice* OR informed decision* OR misinformation OR negotiati* OR nursing role* OR (nurse* AND role*) OR patient acceptance OR patient adherence OR patient attitude* OR patient compliance OR patient cooperation OR patient co-operation OR patient education OR patient involvement OR patient non adherence OR patient noncompliance OR patient nonadherence OR patient non-adherence OR patient noncompliance OR patient non-compliance OR patient participation OR patient preference* OR patient satisfaction OR physician attitude OR physician patient relationship OR physician patient relationships OR physician-patient relationship OR physician-patient relationships OR professional family disagreement* OR professional family relation* OR professional patient disagreement* OR professional-family disagreement* OR professional-family relationship OR professional-family relationships OR professional-patient disagreement* OR psychosocial aspect* OR psychosomatic aspect* OR refusal participat* OR shared decision* OR sharing decision* OR staff attitude* OR treatment refusal* OR uncertainty)

**Search string for all other Databases: CINAHL, PSYCHINFO, Web of Science, Maternity & infant Care Database**

“cesarean section” OR “cesarean sections” OR “caesarean section” OR “caesarean sections” OR “caesarean delivery” OR “caesarean deliveries” OR “cesarean delivery” OR “cesarean deliveries” OR “caesarian delivery” OR “caesarian deliveries” OR “caesarean birth” OR “caesarean births” OR “cesarean birth” OR “cesarean births” OR “caesarian birth” OR “caesarian births” OR “abdominal delivery” OR “abdominal deliveries” OR “surgical birth” OR “surgical births” OR “surgical birthing” OR cesarean OR cesareans OR caesarean OR caesareans OR caesarian OR caesarians OR LSCS OR “C-Section” OR “C-Sections” OR “C Section” OR “C Sections” OR “medicalized childbirth” OR “medicalized childbirths” OR “medicalised childbirth” OR “medicalised childbirths”

AND

clinician OR clinicians OR midwife OR midwives OR obstetrician OR obstetricians OR “obstetric-nurse” OR “obstetric-nurses” OR “obstetric nurse” OR “obstetric nurses” OR “Nurse Midwives” OR “Nurse Midwife” OR “Nurse-Midwife” OR “Nurse-Midwives” OR “care provider” OR “care providers” OR “health care provider” OR “health care providers” OR professional OR professionals OR physician OR physicians OR doctor OR doctors OR “obstetric consultant” OR “obstetric consultants” OR “consultant obstetrician” OR “consultant obstetricians” OR “clinical manager” OR “clinical managers” OR “midwife manager” OR “midwife managers” OR “midwife-manager” OR “midwife-managers” OR “nurse manager” OR “nurse managers” OR “nurse-manager” OR “nurse-managers” OR “certified professional midwife” OR “certified professional midwives” OR “certified nurse-midwife” OR “certified nurse-midwives” OR “certified midwife” OR “certified midwives” OR “consultant midwife” OR “consultant midwives” OR “advanced midwife practitioner” OR “advanced midwife practitioners” OR “clinical midwife specialist” OR “clinical midwife specialists”

AND

experience OR experiences OR experienced OR view OR views OR viewpoint OR viewpoints OR perception OR perceptions OR perceive OR perceived OR attitude OR attitudes OR belief OR beliefs OR perspective OR perspectives OR opinion OR opinions OR concept OR concepts OR thought OR thoughts OR intuition OR awareness OR comprehension OR value OR values OR understanding

AND

“attitude of health personnel” OR “health personnel Attitude” OR “health personnel attitudes” OR “staff attitude” OR “staff attitudes” OR “attitude to health” OR “attitudes to health” OR “health attitude” OR “health attitudes” OR “choice behavior” OR “choice behaviour” OR “choice behaviors” OR “choice behaviours” OR communication OR “personal communication” OR “personal communications” OR communications OR “consumer involvement” OR “consumer participation” OR “consumer participations” OR “cooperative behavior” OR “cooperative behaviors” OR “cooperative behaviour” OR “cooperative behaviours” OR “decision making” OR “decision support technique” OR “decision support techniques” OR “decision theory” OR “decision theories” OR “educational technology” OR “educational technologies” OR “health education” OR “health educations” OR “informed consent” OR “informed consents” OR “professional-family relation” OR “professional-family relationship” OR “professional-family relationships” OR “affective aspect” OR “affective aspects” OR “clinical support technique” OR “clinical support techniques” OR “cognitive aspect” OR “cognitive aspects” OR “collaboration” OR “collaborations” OR “compliant behavio” OR behavior OR behaviors OR behaviour OR behaviours OR behavioral OR behavioural OR behaviorally OR behaviourally OR behaviorism OR behaviorisms OR behaviourism OR behaviourisms OR “acceptance process” OR “acceptance processes” OR consensus OR consumer OR consumers OR consumers' OR consent OR consents OR participation OR participations OR “cooperative behavio” OR “cooperative behavior” OR “cooperative behaviors” OR “co-operative behavio” OR “co-operative behavior” OR “co-operative behaviors” OR “co-operative behaviour” OR “co-operative behaviours” OR decision OR decisional OR decisions OR disput OR disputes OR dissent OR dissents OR “doctor patient relation” OR “doctor patient relations” OR “doctor-patient relation” OR “doctor-patient relationship” OR “doctor-patient relationships” OR “educational technology” OR “educational technologies” OR “emotional aspect” OR “emotional aspects” OR “cognitive aspect”OR “cognitive aspects” OR “psychosomatic aspect” OR “psychosomatic aspects” OR “psychiatric aspect” OR “psychiatric aspects” OR “psychogenic aspect” OR “psychogenic aspects” OR “psychosocial aspect” OR “psychosocial aspects” OR “psycho-social aspect” OR “psycho-social aspects” OR “affective aspect” OR “affective aspects” OR “mental aspect” OR “mental aspects” OR “health education” OR “health educations” OR “health information” OR “health literacy” OR “illness behavio” OR “illness behavior” OR “illness behaviors” OR “illness behaviour” OR “illness behaviours” OR “informed assent” OR “informed assents” OR “informed choice” OR “informed decision” OR “informed decisions” OR “misinformation” OR negotiation OR negotiations OR “nursing role” OR “nursing roles” OR “patient acceptance” OR “patient adherence” OR “patient attitude” OR “patient attitudes” OR “patient compliance” OR “patient cooperation” OR “patient co operations” OR “patient co-operation” OR “patient co-operations” OR “patient education” OR “patient educations” OR “patient involvement” OR “patient non adherence” OR “patient non compliance” OR “patient non adherence” OR “patient non-adherence” OR “patient noncompliance” OR “patient non-compliance” OR “patient participation” OR “patient preference” OR “patient satisfaction” OR “physician attitude” OR “physician attitudes” OR “physician patient relation” OR “physician patient relations” OR “physician-patient relation” OR “physician-patient relationship” OR “physician-patient relationships” OR “professional family disagreement” OR “professional family disagreements” OR “professional patient disagreement” OR “professional patient disagreements” OR “professional-family disagreement” OR “professional-family disagreements” OR “professional-patient disagreement” OR “professional-patient disagreements” OR “refusal to participate” OR “shared decision” OR “shared decisions” OR “sharing decision” OR “sharing decisions”

**Maternity and Infant Care – (using search without inverted commas)**

cesarean section OR cesarean sections OR caesarean section OR caesarean sections OR caesarean delivery OR caesarean deliveries OR cesarean delivery OR cesarean deliveries OR caesarian delivery OR caesarian deliveries OR caesarean birth OR caesarean births OR cesarean birth OR cesarean births OR caesarian birth OR caesarian births OR abdominal delivery OR abdominal deliveries OR surgical birth OR surgical births OR surgical birthing OR cesarean OR cesareans OR caesarean OR caesareans OR caesarian OR caesarians OR LSCS OR C-Section OR C-Sections OR C Section OR C Sections OR medicalized childbirth OR medicalized childbirths OR medicalised childbirth OR medicalised childbirths

AND

clinician OR clinicians OR midwife OR midwives OR obstetrician OR obstetricians OR obstetric-nurse OR obstetric-nurses OR obstetric nurse OR obstetric nurses OR Nurse Midwives OR Nurse Midwife OR Nurse-Midwife OR Nurse-Midwives OR care provider OR care providers OR health care provider OR health care providers OR professional OR professionals OR physician OR physicians OR doctor OR doctors OR obstetric consultant OR obstetric consultants OR consultant obstetrician OR consultant obstetricians OR clinical manager OR clinical managers OR midwife manager OR midwife managers OR midwife-manager OR midwife-managers OR nurse manager OR nurse managers OR nurse-manager OR nurse-managers OR certified professional midwife OR certified professional midwives OR certified nurse-midwife OR certified nurse-midwives OR certified midwife OR certified midwives OR consultant midwife OR consultant midwives OR advanced midwife practitioner OR advanced midwife practitioners OR clinical midwife specialist OR clinical midwife specialists

AND

experience OR experiences OR experienced OR view OR views OR viewpoint OR viewpoints OR perception OR perceptions OR perceive OR perceived OR attitude OR attitudes OR belief OR beliefs OR perspective OR perspectives OR opinion OR opinions OR concept OR concepts OR thought OR thoughts OR intuition OR awareness OR comprehension OR value OR values OR understanding

AND

attitude of health personnel OR health personnel Attitude OR health personnel attitudes OR staff attitude OR staff attitudes OR attitude to health OR attitudes to health OR health attitude OR health attitudes OR choice behavior OR choice behaviour OR choice behaviors OR choice behaviours OR communication OR personal communication OR personal communications OR communications OR consumer involvement OR consumer participation OR consumer participations OR cooperative behavior OR cooperative behaviors OR cooperative behaviour OR cooperative behaviours OR decision making OR decision support technique OR decision support techniques OR decision theory OR decision theories OR educational technology OR educational technologies OR health education OR health educations OR informed consent OR informed consents OR professional-family relation OR professional-family relationship OR professional-family relationships OR affective aspect OR affective aspects OR clinical support technique OR clinical support techniques OR cognitive aspect OR cognitive aspects OR collaboration OR collaborations OR compliant behavio OR behavior OR behaviors OR behaviour OR behaviours OR behavioral OR behavioural OR behaviorally OR behaviourally OR behaviorism OR behaviorisms OR behaviourism OR behaviourisms OR acceptance process OR acceptance processes OR consensus OR consumer OR consumers OR consumers' OR consent OR consents OR participation OR participations OR cooperative behavio OR cooperative behavior OR cooperative behaviors OR co-operative behavio OR co-operative behavior OR co-operative behaviors OR co-operative behaviour OR co-operative behaviours OR decision OR decisional OR decisions OR dispute OR disputes OR dissent OR dissents OR doctor patient relation OR doctor patient relations OR doctor-patient relation OR doctor-patient relationship OR doctor-patient relationships OR educational technology OR educational technologies OR emotional aspect OR emotional aspects OR cognitive aspect OR cognitive aspects OR psychosomatic aspect OR psychosomatic aspects OR psychiatric aspect OR psychiatric aspects OR psychogenic aspect OR psychogenic aspects OR psychosocial aspect OR psychosocial aspects OR psycho-social aspect OR psycho-social aspects OR affective aspect OR affective aspects OR mental aspect OR mental aspects OR health education OR health educations OR health information OR health literacy OR illness behavio OR illness behavior OR illness behaviors OR illness behaviour OR illness behaviours OR informed assent OR informed assents OR informed choice OR informed decision OR informed decisions OR misinformation OR negotiation OR negotiations OR nursing role OR nursing roles OR patient acceptance OR patient adherence OR patient attitude OR patient attitudes OR patient compliance OR patient cooperation OR patient co operations OR patient co-operation OR patient co-operations OR patient education OR patient educations OR patient involvement OR patient non adherence OR patient noncompliance OR patient non adherence OR patient non-adherence OR patient noncompliance OR patient non-compliance OR patient participation OR patient preference OR patient satisfaction OR physician attitude OR physician attitudes OR physician patient relation OR physician patient relations OR physician-patient relation OR physician-patient relationship OR physician-patient relationships OR professional family disagreement OR professional family disagreements OR professional patient disagreement OR professional patient disagreements OR professional-family disagreement OR professional-family disagreements OR professional-patient disagreement OR professional-patient disagreements OR refusal to participate OR shared decision OR shared decisions OR sharing decision OR sharing decisions
