# Supplementary material for: Clinicians’ views of factors influencing decision-making for caesarean section: A systematic review and metasynthesis of qualitative, quantitative and mixed methods studies
Source: PLoS One. 2018 Jul 27;13(7):e0200941. doi: 10.1371/journal.pone.0200941 (PMC6063415; doi:10.1371/journal.pone.0200941)
Supplement: S3 Appendix — (DOCX) [file pone.0200941.s003.docx]

**S3 Appendix - Modified version of the tool for assessment of methodological quality of included studies (Thomas *et al*, 2003)**

| **Quality of study reporting** | **Met the criterion (Score 1)** | **Did not meet the criterion (Score 0)** |
| --- | --- | --- |
| Aims and objectives were clearly reported |  |  |
| Adequate description of context of research |  |  |
| Adequate description of the sample and sampling methods |  |  |
| Adequate description of data collection methods |  |  |
| Adequate description of data analysis methods |  |  |
| **There was good, or some, attempt to establish the:**  Reliability of data collection tools |  |  |
| Validity of data collection tools |  |  |
| Reliability of data analysis |  |  |
| Validity of data analysis |  |  |
| **Quality of methods for research:**  Used appropriate data collection methods to allow for expression of views |  |  |
| Used appropriate methods for ensuring the data analysis was grounded in the views |  |  |
| Actively involved participants in the design and conduct of the study |  |  |
