# Supplementary material for: Clinicians’ views of factors influencing decision-making for caesarean section: A systematic review and metasynthesis of qualitative, quantitative and mixed methods studies
Source: PLoS One. 2018 Jul 27;13(7):e0200941. doi: 10.1371/journal.pone.0200941 (PMC6063415; doi:10.1371/journal.pone.0200941)
Supplement: S4 Appendix — (DOCX) [file pone.0200941.s004.docx]

**S4 Appendix - Results of assessment of methodological quality of included studies**

| **Author(s) and year** | **Aims and objectives were clearly reported** | **Adequate description of context of research** | **Adequate description of the sample and sampling methods** | | **Adequate description of data collection methods** | **Adequate description of data analysis methods** | **Reliability of data collection tools** | **Validity of data collection tools** | **Reliability of data analysis** | **Validity of data analysis** | **Used appropriate data collection methods to allow for expression of views** | **Used appropriate methods for ensuring the analysis was grounded in the views** | **Actively involved participants in the design and conduct of the study** | **Weak (0-6)** | **Moderate (7-9)** | **Strong (10-12)** | |
| --- | --- | --- | --- | --- | --- | --- | --- | --- | --- | --- | --- | --- | --- | --- | --- | --- | --- |
| Appleton *et al* (2000) | √ | √ | | √ | √ | √ | √ | √ | √ | √ |  | √ |  |  |  | 10 |  |
| Arikan *et al* (2011) | √ | √ | | √ | √ | √ |  | √ | √ | √ |  |  |  |  | 8 |  |  |
| Bagheri *et al* (2013) | √ | √ | | √ | √ | √ | √ | √ | √ | √ | √ | √ |  |  |  | 11 |  |
| Bailit *et al* (2007) | √ | √ | | √ | √ | √ |  |  | √ | √ | √ | √ | √ |  |  | 10 |  |
| Bergholt *et al* (2004) | √ | √ | | √ | √ | √ |  |  | √ | √ |  |  |  |  | 7 |  |  |
| Bette *et al* (2007) | √ | √ | | √ | √ | √ |  |  | √ | √ |  |  |  |  | 7 |  |  |
| Bryant *et al* (2007) | √ | √ | | √ | √ | √ | √ | √ | √ | √ | √ | √ |  |  |  | 11 |  |
| Chaillet *et al* (2007) | √ | √ | | √ | √ | √ | √ | √ | √ | √ | √ | √ |  |  |  | 11 |  |
| Chalmers *et al* (1992) | √ | √ | | √ | √ | √ |  |  | √ | √ | √ | √ |  |  | 9 |  |  |
| Chigbu *et al* (2010) | √ | √ | | √ | √ | √ |  | √ |  |  |  | √ |  |  | 7 |  |  |
| Coleman *et al* (2005) | √ | √ | | √ | √ | √ | √ |  | √ | √ | √ |  |  |  | 9 |  |  |
| Coleman-Cowger *et al* (2010) | √ | √ | | √ | √ | √ | √ | √ | √ | √ |  |  | √ |  |  | 10 |  |
| Colomar *et al* (2014) | √ | √ | | √ | √ | √ | √ | √ | √ | √ | √ | √ |  |  |  | 11 |  |
| Cotzias *et al* (2001) | √ | √ | | √ | √ |  |  |  |  | √ | √ | √ |  |  | 7 |  |  |
| Cox (2011) | √ | √ | | √ | √ | √ | √ | √ | √ | √ | √ | √ |  |  |  | 11 |  |
| Danishevski *et al* (2008) | √ | √ | | √ | √ | √ |  | √ |  | √ |  |  |  |  | 7 |  |  |
| Doret *et al* (2010) | √ | √ | | √ | √ | √ |  |  | √ | √ |  |  |  |  | 7 |  |  |
| Faas-Fehervary *et al* (2005) | √ | √ | | √ |  | √ |  |  | √ | √ |  | √ |  |  | 7 |  |  |
| Foureur *et al* (2016) | √ | √ | | √ | √ | √ |  |  | √ | √ | √ | √ |  |  | 9 |  |  |
| Fuglenes and Kristiansen (2009) | √ | √ | | √ | √ | √ |  | √ | √ | √ |  | √ |  |  | 9 |  |  |
| Huang *et al* (2013) | √ | √ | | √ | √ | √ |  | √ | √ | √ |  | √ |  |  | 9 |  |  |
| Josefsson *et al* (2011) | √ | √ | | √ | √ | √ |  |  | √ | √ |  | √ |  |  | 8 |  |  |
| Kabakian-Khasholian *et al* (2007) | √ | √ | | √ | √ | √ |  |  |  |  | √ | √ | √ |  | 8 |  |  |
| Kamal *et al* (2005) | √ | √ | | √ | √ | √ | √ | √ | √ | √ | √ | √ | √ |  |  | 12 |  |
| Karlstrom *et al* (2009) | √ | √ | | √ | √ | √ |  |  | √ | √ | √ | √ | √ |  |  | 10 |  |
| Kenton *et al* (2005) | √ | √ | | √ | √ | √ |  |  | √ | √ | √ | √ |  |  | 9 |  |  |
| Koigi-Kamau *et al* (2005) | √ | √ | | √ | √ | √ |  |  |  |  | √ | √ | √ |  | 8 |  |  |
| Kwee *et al* (2004) | √ | √ | | √ | √ | √ |  |  | √ | √ | √ | √ | √ |  |  | 10 |  |
| Litorp *et al* (2015a) | √ | √ | | √ | √ | √ |  |  | √ | √ | √ | √ | √ |  |  | 10 |  |
| Litorp *et al* (2015b) | √ | √ | | √ | √ | √ |  |  | √ | √ | √ | √ | √ |  |  | 10 |  |
| Monari *et al* (2008) | √ | √ | | √ | √ | √ | √ | √ | √ | √ | √ | √ |  |  |  | 11 |  |
| Samadi *et al* (2013) | √ | √ | | √ | √ | √ | √ | √ |  |  | √ |  |  |  | 8 |  |  |
| Weaver *et al* (2007) | √ | √ | | √ | √ | √ |  |  | √ | √ | √ | √ | √ |  |  | 10 |  |
| Yazdizadeh *et al* (2011) | √ | √ | | √ | √ | √ |  |  | √ | √ | √ | √ |  |  | 9 |  |  |
