# Supplementary material for: Clinicians’ views of factors influencing decision-making for caesarean section: A systematic review and metasynthesis of qualitative, quantitative and mixed methods studies
Source: PLoS One. 2018 Jul 27;13(7):e0200941. doi: 10.1371/journal.pone.0200941 (PMC6063415; doi:10.1371/journal.pone.0200941)
Supplement: S5 Appendix — (DOCX) [file pone.0200941.s005.docx]

**S5 Appendix – Studies conducted in OECD and Non-OECD countries**

| **Author(s)/Year** | **Study location** | **OECD/Non-OECD** | **Number of studies** |
| --- | --- | --- | --- |
| Bailit *et al* (2007); Bettes *et al* (2007); Coleman *et al* (2005); Coleman-Cowger *et al* (2010); Colomar *et al* (2014); Cox (2011); Kenton *et al* (2005) | US | OECD | 7 |
| Cotzias *et al* (2001); Kamal *et al* (2005); Weaver and Richards (2007) | UK | OECD | 3 |
| Appleton *et al* (2000); Bryant *et al* (2007); Foureur *et al* (2016) | Australia | OECD | 3 |
| Josefsson *et al* (2011); Karlstrom *et al* (2009) | Sweden | OECD | 2 |
| Chaillet *et al* (2007) | Canada | OECD | 1 |
| Arikan *et al* (2011) | Turkey | OECD | 1 |
| Bergholt *et al* (2004) | Denmark | OECD | 1 |
| Doret *et al* (2010) | France | OECD | 1 |
| Faas-Fehervary *et al* (2005) | Germany | OECD | 1 |
| Fuglenes and Kristiansen (2009) | Norway | OECD | 1 |
| Kwee *et al* (2004) | Netherlands | OECD | 1 |
| Monari *et al* (2008) | Italy | OECD | 1 |
| Bagheri *et al* (2013); Samadi *et al* (2013); Yazdizadeh *et al* (2011) | Iran | Non-OECD | 3 |
| Litorp *et al* (2015a); Litorp *et al* (2015b) | Tanzania | Non-OECD | 2 |
| Chalmers *et al* (1992) | South Africa | Non-OECD | 1 |
| Chigbu *et al* (2010) | Nigeria | Non-OECD | 1 |
| Danishevski *et al* (2008) | Russia | Non-OECD | 1 |
| Huang *et al* (2013) | China | Non-OECD | 1 |
| Kabakian-Khasholian *et al* (2007) | Lebanon | Non-OECD | 1 |
| Koigi-Kamau and Kiarie (2005) | Kenya | Non-OECD | 1 |
