# Supplementary material for: Clinicians’ views of factors influencing decision-making for caesarean section: A systematic review and metasynthesis of qualitative, quantitative and mixed methods studies
Source: PLoS One. 2018 Jul 27;13(7):e0200941. doi: 10.1371/journal.pone.0200941 (PMC6063415; doi:10.1371/journal.pone.0200941)
Supplement: S6 Appendix — (DOCX) [file pone.0200941.s006.docx]

**S6 Appendix Issues within a cultural context (similarities and differences) - OECD versus Non-OECD countries**

| **Key issues within cultural context - OECD countries** | **Key issues within cultural context - Non-OECD countries** |
| --- | --- |
| **Similarities in views among clinicians from OECD and Non-OECD countries** | |
| **Women’s request for CS**  Clinicians from OECD countries believed in women’s right to choose a CS.  *“At the end of the day, I feel very strongly that women, at the end of the day it’s their body and it’s their right to choose. And I certainly feel that as long as it’s an informed consent, I would be very agreeable to obliging either way.” (Obstetrician) (Bryant et al, 2007 p.1194) [18]*  Women’s perceived fear was viewed to be a reason for their request for CS.  *“There are a lot of women who are afraid of everything. They have no trust in their bodily functions or that we are made to give birth.” (Focus group discussion with midwives and obstetricians) (Karlstrom et al, 2009 p. 60) [29]* | **Women’s request for CS**  Similar to OECD countries, clinicians from Non-OECD countries believed on women’s right to choose a CS.  *“I tell them all the advantages and disadvantages and a complication of caesarean section, but this is the mother, who should choose the type of delivery.” (Obstetrician) (Bagheri et al, 2013 p.46) [[14]*  *“Natural birth is painful. Sometimes they have pain for 24 hours... Some have negative experiences from their previous deliveries. They might have a difficult one... When we tell them that second delivery is much easier they don’t believe us, and if we resist, they go to another doctor.” (Obstetrician) (Bagheri et al, 2013 p.46) [14]* |
| **CS being a ‘safe option’**  Clinicians from OECD countries believed CS to be a ‘safe option’.  *“Elective caesarean sections I view as being quite safe. Emergency caesarean sections, because you’re rushing, and may be … a bit more dangerous, although still it’s a relatively safe operation.” (Obstetrician) (Bryant et al, 2007 p.1197) [18]* | **CS being a ‘safe option’**  Similar to OECD countries, clinicians from Non-OECD countries believed CS to be a ‘safe option’.  *“Earlier on, CS was very dangerous in our setting. Nowadays that we feel that CS is safe, we tend to do more CSs.” (Senior obstetrician) (Litorp et al, 2015a p.717) [49]* |
| **Personal convenience**  CS was viewed to be a convenient option.  *“It is certainly easier to do a repeat C-section, so why not just say, ‘Shoot, I don’t have to deal with VBACs, great…and I get to have a little bit of easier life.’ I think when you get to the heart of it, that’s what’s going on.” (Obstetrician) (Cox, 2011. p.6) [17]* | **Personal convenience**  CS was viewed to a convenient option.  *“We should manage our work. The caesarean section gives us the opportunity to manage our schedules, finding someone to work instead of us, tell the hospital when we are leaving. Of course, physicians welcome this”. (Obstetrician) (Bagheri et al, 2013 p.e47) [14]*  *“With CS I minimize my time and I earn more!” (Obstetrician) (Litorp et al, 2015b) [19]* |
| **Differences in views among clinicians from OECD and Non-OECD countries** | |
| **Litigation (fear of adverse outcome and related legal consequences)**  Fear of adverse outcome and subsequent litigation was a major issue among clinicians from OECD countries.  *“...sometimes you feel fearful about the outcome, like the old primipara with her fifth IVF treatment. You feel nothing must go wrong and wouldn’t it be better with a CS just in case.” (Obstetrician) (Karlstrom et al, 2009, Sweden p. 60)[29]*  *“I just think it’s a bunch of crap that you have to change your practice when you know something is safe because somebody might sue you. Anytime you get a less than optimal outcome, people want to blame, people want to sue... It’s just kind of a personal philosophy, too. I just think that most long-term midwives get to that point. Otherwise you’d be too afraid to do anything. Birth is amazing, and not always predictable.” (Midwife) (Cox 2011, p. 5) [17]* | **Litigation (social stigma)**  Social stigma associated with litigation was a major concern among clinicians from Non-OECD countries.  *“Being brought to the court, even once, makes the physician and her near friends keep away from vaginal deliveries for ever. In the court they behave rudely towards the physician, making her behave in a similar manner towards others.” (Obstetrician) (Yazdizadeh et al, 2011 p.5)[13]* |
| **Resources (staff shortages and work load related stress)**  Staff shortages and workload-related stress were issues among clinicians from OECD countries.  *“The major rise in the CS rate in Sweden is due to stress in the delivery units.”(midwife) (Karlstrom et al, 2009 p. 60)[29]* | **Resources (physical and man power resources)**  Lack of infrastructure and physical resources were issues among clinicians from Non-OECD countries.    *“Our centre is too crowded and this is an important factor. We send expectant mothers who can be C-sectioned rapidly to the operation room in order to have more vacant beds.”(Midwife*) *(Yazdizadeh et al, 2011.p.7)[13]*  In Chalmer *et al*’s study, 15% (n=35 of 233) of obstetricians stated lack of access to facilities influenced their decision to perform CS [36]. |
| **Private versus public system**  Difference in practice among private and public sectors, and possible influence of financial factors, were some concerns among clinicians from OECD countries.  Obstetricians working in private hospitals were reported to perform CS on maternal request at a significantly higher level than those working in public hospitals (Obstetrician) (Arikan et al, 2011) [5]  *“In the private sector, providers are reimbursed approximately $700 for normal childbirth and $1500 for caesarean section, so the doctor prefers to perform caesarean.” (Obstetricians) (Colomar et al, 2014. P.2388)[11]* | **Insurance and payment issues**  Issues related to insurance system and related financial matters were concerns among clinicians from Non-OECD countries.  *“In Iran, the insurance companies sign a contract with healthcare providers and pay them rather than compensating the service itself. Considering the fact that the service provided by the midwives is not covered by insurance companies, expectant moms prefer to go to a specialist. In this situation the rate of additional interventions and C-sections would increase.” (Midwife) (Yazdizadeh et al, 2011 p.4)[13]*  The payment system to obstetricians was viewed as a factor.  *“Many midwives claim that physicians receive all the money so why should a midwife spend long hours in the labor room; physicians, on the other hand, claim they should receive more money as they are in charge of any possible legal problems linked to labor.” (Obstetrician) (Yazdizadeh et al, 2011 p.4)[13]* |
