# Supplementary figures and images for: Clinicians’ views of factors influencing decision-making for caesarean section: A systematic review and metasynthesis of qualitative, quantitative and mixed methods studies
Source: PLoS One. 2018 Jul 27;13(7):e0200941. doi: 10.1371/journal.pone.0200941 (PMC6063415; doi:10.1371/journal.pone.0200941)

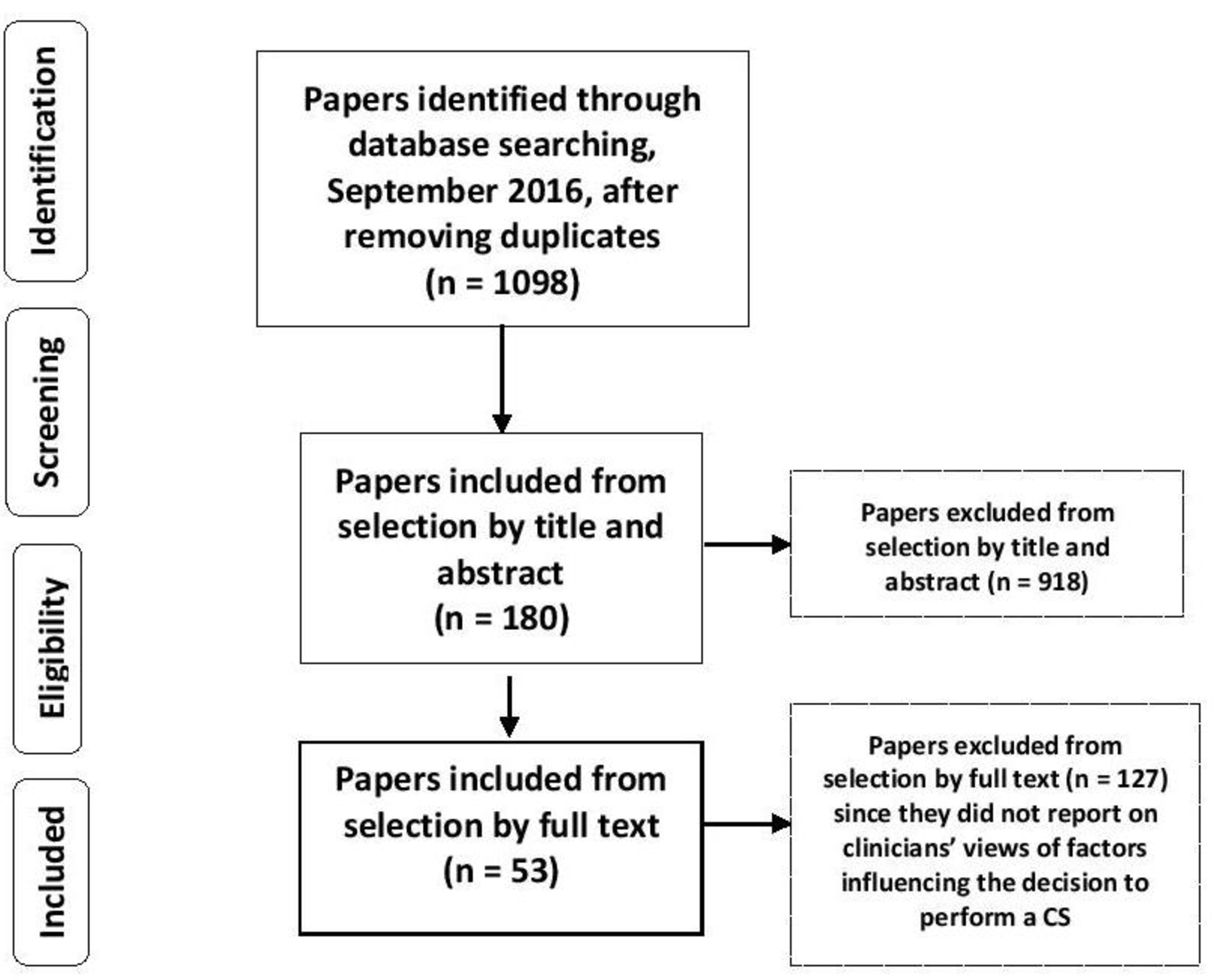

Supplement: S1 Fig — (TIF) [file pone.0200941.s007.tif]

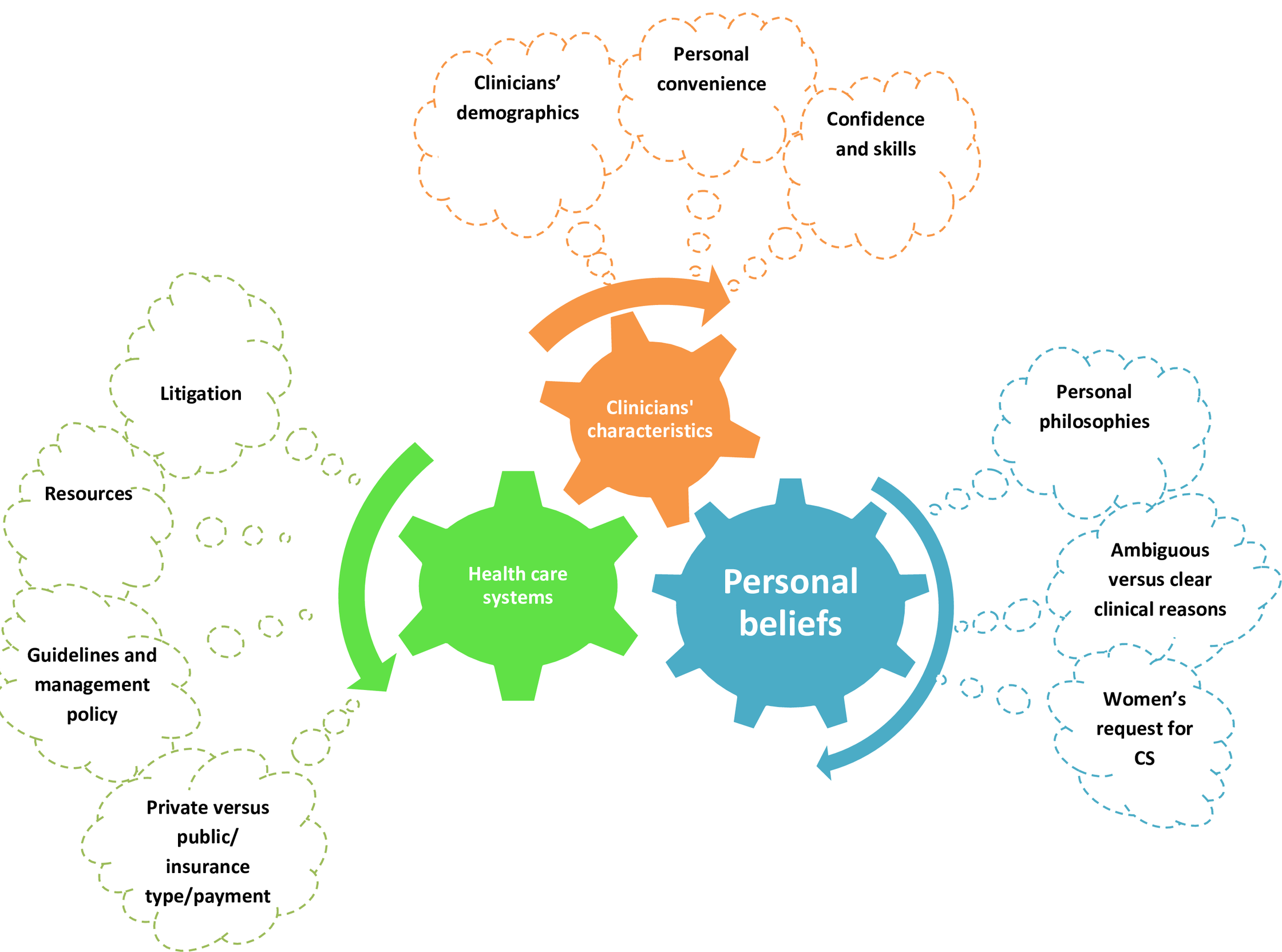

Supplement: S2 Fig — (TIF) [file pone.0200941.s008.tif]
